# Development and Verification of the Amino Metabolism-Related and Immune-Associated Prognosis Signature in Gliomas

**DOI:** 10.3389/fonc.2021.774332

**Published:** 2021-11-05

**Authors:** Yang Xu, Liguo Ye, Rongxin Geng, Ping Hu, Qian Sun, Shiao Tong, Fanen Yuan, Qianxue Chen

**Affiliations:** Department of Neurosurgery, Renmin Hospital of Wuhan University, Wuhan, China

**Keywords:** gliomas, gene signature, amino acid metabolism, prognosis, microenvironment, immune

## Abstract

Aberrant reprogramming of metabolism has been considered a hallmark in various malignant tumors. The metabolic changes of amino acid not only have dramatic effects in cancer cells but also influence their immune-microenvironment in gliomas. However, the features of the amino acid metabolism-related and immune-associated gene set have not been systematically described. The expression level of mRNA was obtained from The Cancer Genome Atlas database and the Chinese Glioma Genome Atlas database, which were used as training set and validation set, respectively. Different bioinformatics and statistical methods were combined to construct a robust amino metabolism-related and immune-associated risk signature for distinguishing prognosis and clinical pathology features. Constructing the nomogram enhanced risk stratification and quantified risk assessment based on our gene model. Besides this, the biological mechanism related to the risk score was investigated by gene set enrichment analysis. Hub genes of risk signature were identified by the protein–protein interaction network. The amino acid metabolism-related and immune-associated gene signature recognized high-risk patients, defined as an independent risk factor for overall survival. The nomogram exhibited a high accuracy in predicting the overall survival rate for glioma patients. Furthermore, the high risk score hinted an immunosuppressive microenvironment and a lower sensitivity of immune checkpoint blockade therapy and also identified PSMC5 and PSMD3 as novel biomarkers in glioma. In conclusion, a novel amino acid metabolism-related and immune-associated risk signature for predicting prognosis in glioma has been constructed and identified as two potential novel biomarkers.

## Introduction

Metabolic reprogramming is critical for maintaining the survival of cancer cells and defined as a hallmark of cancer, which might be the consequence of oncogenic mutations (1). Amino acid metabolism also emerges as an important role in the metabolic reprogramming of cancer cells because of its function in redox balance, energy regulation, biosynthesis support, and so on (2). Amino acids and their derivatives can not only regulate cancer cells but also modulate the surrounding microenvironment, which enhances the malignancy and immunosuppression of tumors (3), for instance, arginine derivations could change the chromatin structure to regulate gene expression, which promotes the proliferation of cancer cells (4). Kynurenine, which is the catabolic product of tryptophan, induces the invasion of cancer cells and the immunosuppression of a tumor microenvironment (5) by binding to transcription factor aryl hydrocarbon receptor (AHR) (6). Moreover, the activation of AHR hampers the performance of dendritic cells and T cells, which play a role in anti-tumor (7). The metabolism of amino acid is varied in tumors and plays a significant role not only in the biological process of tumor cells but also in the tumor microenvironment, particularly the modulation of the immune. All these indicate a better understanding of the metabolism of amino acids, which will offer potentially effective targets for cancer therapy (8).

In our study, we focus on glioma which is a group of highly heterogeneous neurocutaneous tumors, accounting for about 26% of all intracranial tumors, and is the most deadly primary malignant type of brain tumor in adults (9). Although combined therapy has been developed, including precise surgical resection, adjuvant radiotherapy, and temozolomide chemotherapy, the overall survival remains poor and has not been significantly improved. Furthermore, long-term survival is rare (10). Although immunotherapy has made a breakthrough progress in the treatment of a variety of solid tumors, the specific effect of immunotherapy in glioma is still not clear (11). It has been found that the expression level of immunosuppressive factors such as PD-L1 and IDO/TDO were dramatically elevated in gliomas. As is well known, PD-L1 could limit the function of effect T cells, and the metabolite mediated by IDO/TDO promotes the development of an immunosuppressive microenvironment (12, 13). In addition, the upregulation of Treg cells could exhausting cytotoxic T cells to reduce the damage of tumor cells and enhance the immunosuppressive effects in the glioma microenvironment (14). However, how amino acid metabolism influences prognosis and the immune process in glioma progression needs further systematic research.

In our study, the amino acid metabolism-related and immune-associated risk signature was defined as an independent risk factor for the prognosis of glioma patients. The decision tree strongly verified the risk-dependent subgroups, and the nomogram showed an extremely high accuracy. In addition, a high risk score hinted an immunosuppressive microenvironment and lower sensitivity of ICB therapy, and PSMC5 and PSMD3 were identified as novel biomarkers in glioma. In summary, we demonstrated a novel amino acid metabolism-related and immune-associated risk signature for predicting prognosis in patients with glioma and identified two potential novel biomarkers.

## Materials and Methods

**Figure 1:**
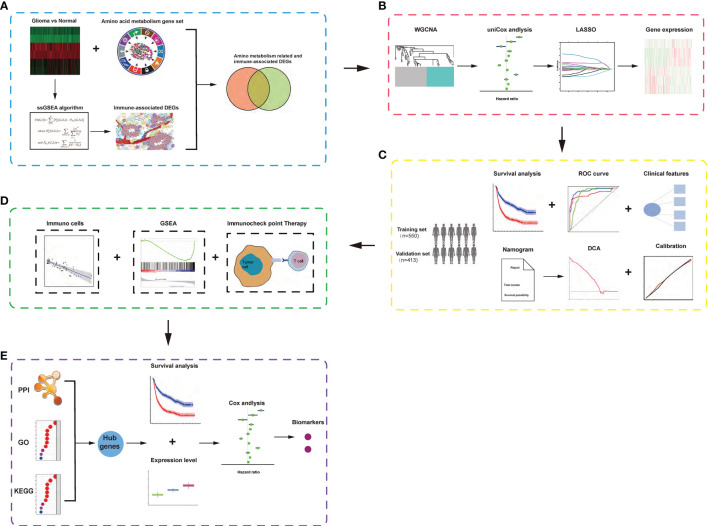
Schematic diagram of the study design. **(A)** Identification of amino acid metabolism-related and immune-associated gene module in glioma patients among various hallmarks of cancer. **(B)** WGCNA and least absolute shrinkage and selection operator Cox algorithms were combined to develop an amino acid metabolism-related and immune-associated gene signature for prognosis. **(C)** The prognostic and predictive capacities were validated in different cohorts and methods. **(D)** Comprehensive analyses of enriched pathways, immune cell infiltration, and therapeutic responses in different risk groups. **(E)** Identification of hub genes and biomarkers from gene signature for glioma.

The workflow of our analysis is shown in **Figure 1**, and specific details are explained in the following sub-sections.

### Data Preparation and Collection

The expression of mRNA and the clinical information of patients were collected from 698 patients in the cancer genome atlas database. Consistently, 413 samples were collected from 693 samples in the Chinese Glioma Genome Atlas, part B, as a validation set. Moreover, the mRNA data of normal brain tissue was collected from the Genotype–Tissue Expression Project (GTEx).

### Obtaining Amino Metabolism-Related Genes

Amino acid metabolism-related gene sets (REACTOME_ METABOLISM_OF_AMINO_ACIDS_AND_DERIVATIVES) were obtained from the Molecular Signatures Database, v5.1 (MSigDB) (http://www.broad.mit.edu/gsea/msigdb/).

### Determination of the Immune Status Through Single-Sample Gene Set Enrichment Analysis

Using the single-sample Gene Sets Enrichment Analysis (ssGSEA) algorithm based on the transcriptome profiling data and corresponding immunity-related gene sets retrieved from MSigDB (15, 16) and using ESTIMATE algorithm, we analyzed the estimation of stromal and immune cells in tumor tissues (17), which has been developed to measure stromal level (stromal score), cyto-infiltration degree (immune score), and tumor purity.

### Construction of Amino Metabolism-Related and Immune-Associated Signatures

The R package “WGCNA” was used to construct a scale-free co-expression network to verify a gene module that is mostly related to amino metabolism and immune in glioma. To explore the most robust genes, the LASSO regression model was performed (18). Furthermore, the risk scores were calculated by multiplying gene expression by the regression coefficient acquired upon Lasso regression. Based on the median risk score, all cases were divided into high- or low-risk groups.

### Prognostic Value and TIC Profile of the Risk Model

The prognostic significance of the risk signature was evaluated by Kaplan–Meier survival curves. Independent prognostic factors, including the risk score in glioma, were investigated by univariate and multivariate Cox regression analyses. Subsequently, we investigated the specificity and sensitivity of risk score in the prediction of 5-year overall survival (OS) by analyzing the receiver operating characteristic (ROC) curve (19). Next, a nomogram according to related prognostic factors was constructed to quantitatively predict the 1-, 2-, and 3-year survival rate in glioma patients. The CIBERSORT package in R was used to evaluate differences in the frequencies of 22 immune cell types (including Tregs and CD4+ cells) in glioma. The CIBERSORT is widely used in evaluating the type of immune cell in the microenvironment through estimating relative subsets of known RNA transcript (CIBERSORT) software (https://cibersort.stanford.edu/) (20). The computational method was used in the low- and high-risk groups to explore the correlation of the TICs in different groups.

### Gene Set Enrichment Analysis

In the Molecular Signatures Database, Hallmark and C7 gene sets were downloaded, which were used as the target gene sets to investigate the gene sets associated with risk score in the whole transcriptome of all glioma samples in the TCGA performed by the software GSEA-3.0. (NOM *p* < 0.05 and FDR *q <*0.05 were considered significant).

### Functional Annotation for Genes of Interest and Construction of PPI

To explore the gene ontology (GO) of selected genes, R package cluster Profiler package was used to explore the functions among genes of interest, with a cutoff criterion of adjusted *p <*0.05. The GO annotation that contains the three sub-ontologies— biological process, cellular component (CC), and molecular function—can identify the biological properties of genes and gene sets for all organisms (21). The Online tool Search Tool for the Retrieval of Interacting Genes (STRING) was used to predict protein–protein interactions (PPI) and construct a PPI network of selected genes. Using the STRING database, genes with a score of 0.4 were chosen to build a network model visualized by Cytoscape (v3.7.2) (22). In a co-expression network, Maximal Clique Centrality (MCC) algorithm was reported to be the most effective method of finding hub nodes (19). The MCC of each node was calculated by CytoHubba, a plugin in Cytoscape (23). In this study, the genes with the top 10 MCC values were considered hub genes.

### Verification of the Expression Patterns and the Prognostic Values of Hub Genes

To explore the potential reliability of the hub genes, the expression level of each hub gene between cancer and normal tissue was plotted as a box plot graph. Based on the TCGA database, Kaplan–Meier univariate survival analysis was performed by using the survival package in R software to explore the relationship between overall survival and disease-free survival with hub genes in patients. In the study, all the patients selected for survival analysis should be with complete clinical information. Consequently, based on the median expression value of hub genes, these samples were divided into two subgroups. The survival-related hub genes with log-rank *p <*0.05 were regarded as statistically significant.

### Human Tissue Samples

Normal brain tissues were collected from patients who suffered from a serious brain injury. The glioma samples were obtained from the Department of Neurosurgery, Renmin Hospital of Wuhan University, Wuhan, China. The clinical glioma specimens were examined and diagnosed by pathologists at Renmin Hospital of Wuhan University. This study was approved by the Institutional Ethics Committee of the Faculty of Medicine at Renmin Hospital of Wuhan University [approval number: 2012LKSZ (010) H]. Informed consent was obtained from all patients whose tissues were used.

### RNA Extraction and Quantitative Real-Time PCR

Total RNA of glioma tissues was extracted using Trizol reagent (G3013-100ML, Servicebio, Wuhan, China), and cDNA was synthesized by SweScript RT I First Strand cDNA Synthesis Kit (G3330-50; Servicebio, Wuhan, China). Quantitative real-time PCR (qPCR) for PSMC3 and PSMD5 mRNA levels were performed using SYBR qPCR SuperMix (E096-01B, Novoprotein, China) according to the instructions of the manufacturer and performed in Bio-Rad CFX Manager 2.1 real-time PCR Systems (Bio-Rad, Hercules, CA, USA).

GAPDH was set as internal control, and the relative Ct method was used to analyze the data. The sequences of primers are listed in **Supplementary Table S3**.

## Results

### Construction of Weighted Gene Co-expression Modules

First, combining the mRNA data in TCGA glioma and GTEx database, 10,550 differentially expressed genes between glioma and normal brain tissue were detected. Among these genes, 260 amino acid metabolism-related genes were ensured (**Supplementary Figure S1A**). The KEGG and GO analyses confirmed that these nods were mainly related to the biological function and pathway of amino acid metabolism. The PPI network showed a strong co-expressed correlation among the genes (**Supplementary Figures S1B–D**). Then, the samples in the training set were hierarchically clustered in the immunity-high (immunity-H) or immunity-low (immunity-L) group by ssGSEA (**Supplementary Figures S2A, B** **)**. The box plot of the fraction of immune cells in glioma tissues was significantly different among immunity-H and immunity-L groups (**Supplementary Figure S2C**). Consistently, the stromal scores, immune scores, and ESTIMATE scores of glioma samples in the immunity-H group remarkably increased compared with those in the immunity-L group (**Supplementary Figures S2D, F** **)**. Meanwhile, the tumor purity in the immunity-H group was significantly lower than in the immunity-L group (**Supplementary Figure S2G**). Besides this, patients in the immunity-H group had a significantly poorer prognosis than those in the other groups (**Supplementary Figure S2H**).

**Figure 2:**
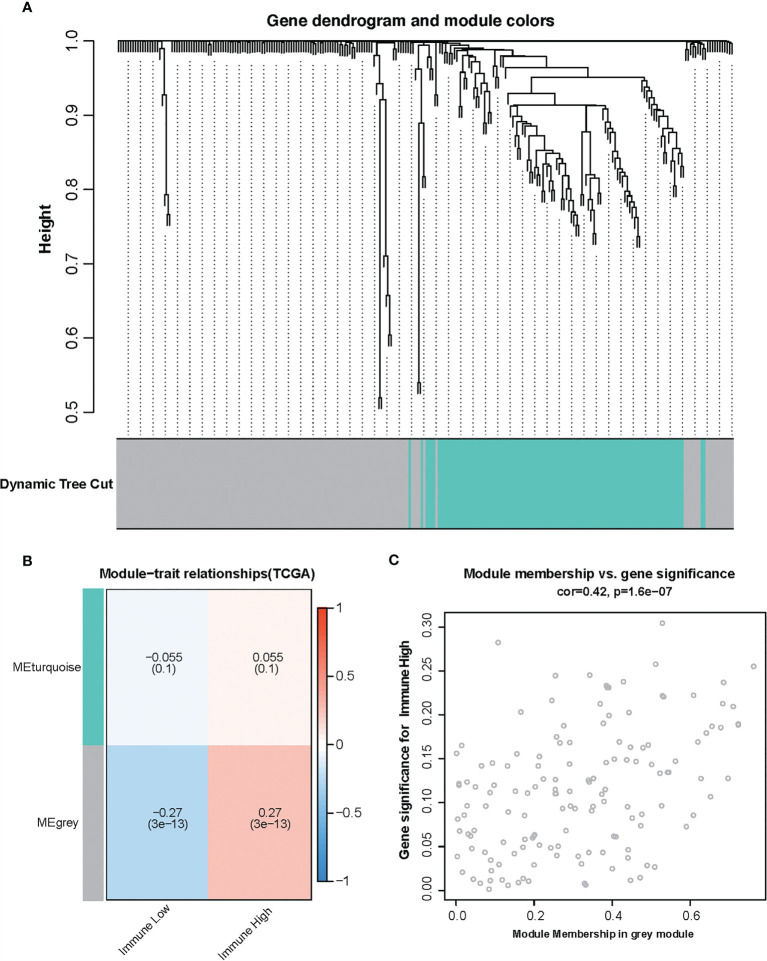
Identification of modules associated with the immunity in the Cancer Genome Atlas glioma dataset. **(A)** The cluster dendrogram of co-expression network modules was ordered by a hierarchical clustering of genes based on the 1-TOM matrix. Each module was assigned different colors. **(B)** Module–immune relationships. Each row corresponds to a color module, and each column corresponds to immune score (high and low). **(C)** Module membership *vs*. gene significance in gray module.

To find the correlation between amino acid metabolism-related genes and immune infiltration in TCGA glioma, gene co-expression networks were constructed from the TCGA glioma datasets with the WGCNA package. Two modules in the TCGA glioma were recognized, and a different color was assigned for each module (**Figure 2A**). Then, we created a heat map of module–immune relationships to evaluate the association between each module and different immune scores (high and low). The results of the module–immune relationships showed that the gray module had the highest association with the immune-high group (pink module: *r* = 0.27, *p* < 0.001) in TCGA glioma (**Figure 2B**). The module membership and gene significance were highly correlated in the gray module (**Figure 2C**).

### Identification of a 12-Gene Risk Signature Associated With Amino Acid Metabolism and Immune in Glioma

**Figure 3:**
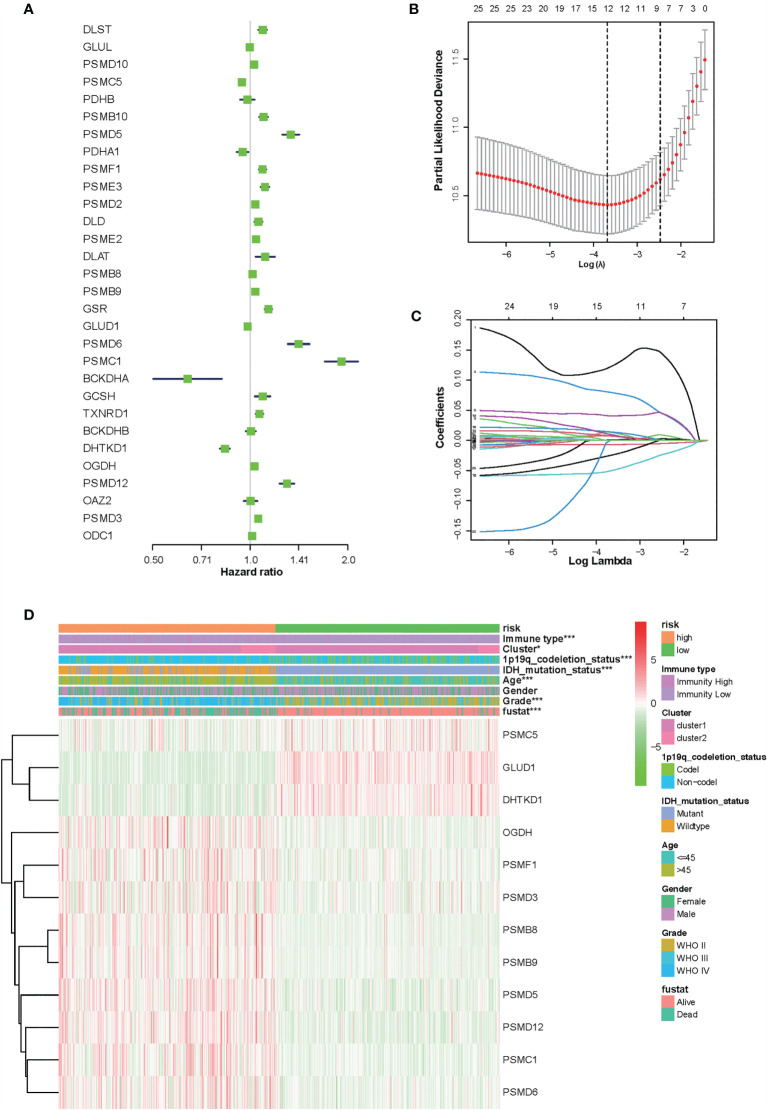
Identification of 12-gene risk signature for overall survival (OS) by least absolute shrinkage and selection operator regression analysis in the Chinese Glioma Genome Atlas datasets. **(A)** Thirty genes associated with OS of patients with glioma by univariate Cox regression analysis. **(B)** Red dots represent average partial likelihood deviances for every model with a given lambda, and vertical bars indicate the upper and lower values of the partial likelihood deviance errors. The vertical black dotted lines define the optimal values of lambda, which provides the best fit. Survival curves of patients in the high-risk group and the low risk group of The Cancer Genome Atlas glioma cohort. **(C)** The selection of the tuning parameter (lambda) in the least absolute shrinkage and selection operator model by 10-fold cross-validation based on minimum criteria for OS; the lower X axis shows log (lambda), and the upper X axis shows the average number of OS genes. The Y axis indicates partial likelihood deviance error. **(D)** Heat map showing the association of risk scores and clinical pathology features based on the 12-gene risk signature. *P < 0.05, ***P < 0.001.

To identify the amino acid metabolism-related and immuno-associated risk signature, the univariate Cox regression analysis was used to select 30 genes in the training set, which were related to the prognosis of patients (**Figure 3A**). Thereafter, the most relevant biomarkers for prognosis were identified through the LASSO Cox regression model, and overfitting was counteracted by 10-fold cross-validation. As a result, the group of 12 genes (PSMC5, GLUD1, DHTKD1, OGDH, PSMF1, PSMD3, PSMB8, PSMB9, PSMD5, PSMD12, PSMC1, and PSMD6) was extracted according to LASSO coefficients (**Figures 3B, C** **)**. The median risk score was defined as the cutoff value to divide the training set into two subgroups, including high- and low-risk groups, and a significant difference was found in both the molecular and clinical characteristics between these subgroups (**Figure 3D**).

**Figure 4:**
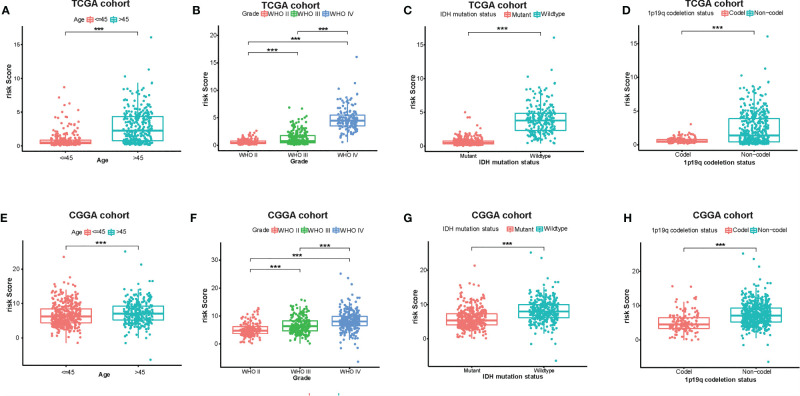
Associations between the amino acid-related and immune-associated signature and other features in both the TCGA and CGGA datasets. Distribution of the amino acid-related and immune-associated gene signature in patients stratified by age **(A**, **E)**, WHO grade **(B**, **F)**, IDH1 status in each grade **(C**, **G)**, and 1p/19q status **(D**, **H)**; ***P < 0.001. IDH, isocitrate dehydrogenase; TCGA, The Cancer Genome Atlas; CGGA, Chinese Glioma Genome Atlas; Codel, codeletion; GBM, glioblastoma; WHO, World Health Organization.

At the same time, there were significant differences according to the risk signature values of age-stratified and WHO-grade-stratified clinical samples in both the TCGA and CGGA cohorts (**Figures 4A, B, E, F** **)**. The molecular pathological diagnosis of glioma has been put forward in clinical practice. IDH wild type and 1p19q non-codeletion gliomas were all the poor prognostic factors and had an inadequate response to traditional radiotherapy or chemotherapy of glioma patients (24). Such being the case, the distribution of the 12-gene signature was explored based on IDH status-stratified clinical samples (**Figures 4C, G** **)** and 1p/19q codeletion status (**Figures 4D, H** **)**. Overall, these results indicated that the risk score based on the gene signature was significantly associated with clinical features.

### Development of the Risk Score Signature and Assessment of the Predicting Capacity

**Figure 5:**
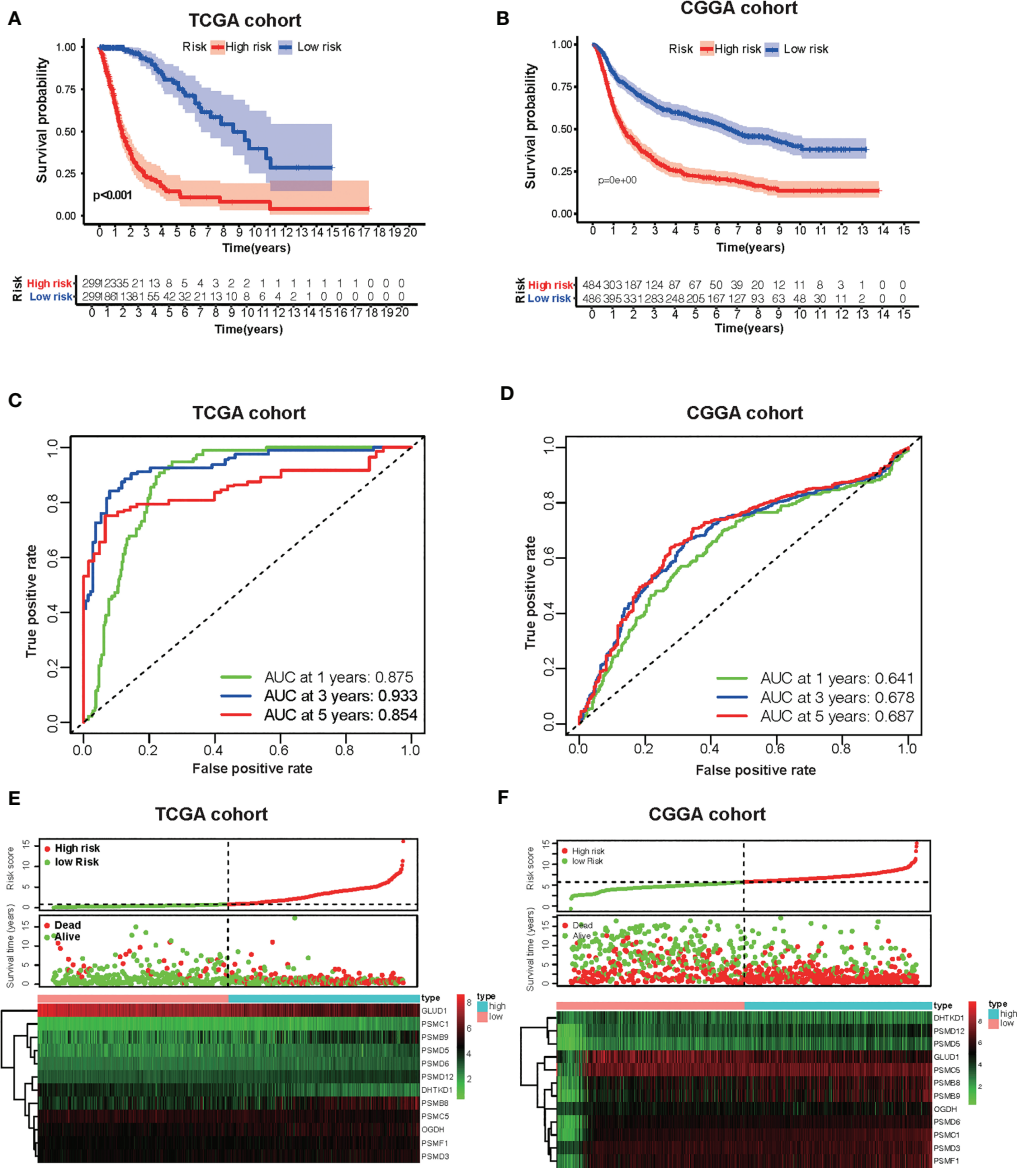
Development of the risk score signature and assessment of the predicting capacity. **(A, B)** Survival curves of patients in the high-risk group and the low-risk group of The Cancer Genome Atlas (TCGA) glioma and Chinese Glioma Genome Atlas (CGGA). Patients in the high-risk group suffered shorter overall survival. **(C, D)** Survival-dependent receiver operating characteristic (ROC) curves validation at 1, 3, and 5 years of prognostic value of the prognostic index in the two databases (TCGA and CGGA, respectively). **(E, F)** The distribution of risk score, overall survival (OS), gene expression in TCGA, and CGGA databases is also shown. The distribution of risk score, OS, and heat map of the expression of 12 genes in the low- and high-risk groups are shown in the picture from top to bottom.

Based on the groups with high and low risk scores, Kaplan–Meier analysis was performed, and it showed that patients with high risk scores had dramatically reduced overall survival compared with patients with a low risk score in both TCGA and CGGA datasets (**Figures 5A, B** **)**. Besides this, as far as the 1-, 3-, and 5-year overall survival is in question, the values of the area under the curve (AUC) of the ROC curve for the TCGA glioma cohort were 0.875, 0.933, and 0.854. Consistently, concerning 1-, 3-, and 5-year overall survival, the values of AUC for the CGGA cohort were 0.641, 0.678, and 0.687, respectively (**Figures 5C, D** **)**. Furthermore, the plots were listed to show the distribution of gene expression, risk score, and survival status basing on the amino acid metabolism- and immune-related signature in TCGA and CGGA (**Figures 5E, F** **)**. To further explore the significance of our model in evaluating prognosis independently, we performed a univariate analysis as well as a multivariate analysis, which showed that the value of the risk score might be defined as an independent factor to evaluate the prognosis of glioma patients in both TCGA and CGGA (**Table 1**).

**Table 1:** Univariate and multivariate Cox regression analyses of clinicopathologic characteristics associated with overall survival in TCGA dataset and CGGA dataset.

Variables	TCGA Dataset						CGGA Dataset					
	Univariate analysis			Multivariate analysis			Univariate analysis			Multivariate analysis		
	HR	95%CI	P-value	HR	95%CI	*P*-value	HR	95%CI	*P*-value	HR	95%CI	*P*-value
**Grade**	4.987	3.873–6.421	<0.001	2.059	1.530–2.772	<0.001	2.635	2.353–2.952	<0.001	2.079	1.820–2.374	<0.001
**Gender**	1.011	0.747–1.368	0.944	0.974	0.710–1.336	0.870	1.022	0.870–1.202	0.787	1.020	0.866–1.200	0.815
**Age**	4.863	3.391–6.975	<0.001	2.296	1.465–3.596	<0.001	1.922	1.638–2.256	<0.001	1.227	1.033–1.457	0.020
**IDH mutation status**	0.090	0.063–0.129	<0.001	0.553	0.306–1.001	<0.051	0.367	0.315–0.428	<0.001	0.657	0.558–0.773	<0.001
**1p19q codeletion status**	0.217	0.128–0.370	<0.001	0.543	0.287–1.025	0.060	0.527	0.447–0.621	<0.001	0.781	0.661–0.923	0.004
**Risk score**	9.425	6.355–13.978	<0.001	2.672	1.510–4.729	<0.001	2.448	2.076–2.887	<0.001	1.364	1.131–1.646	0.001

HR, hazard ratio; CI, confidence interval.

### Combination of the Risk Signature and Clinicopathological Features Improves Risk Stratification and Survival Prediction

**Figure 6:**
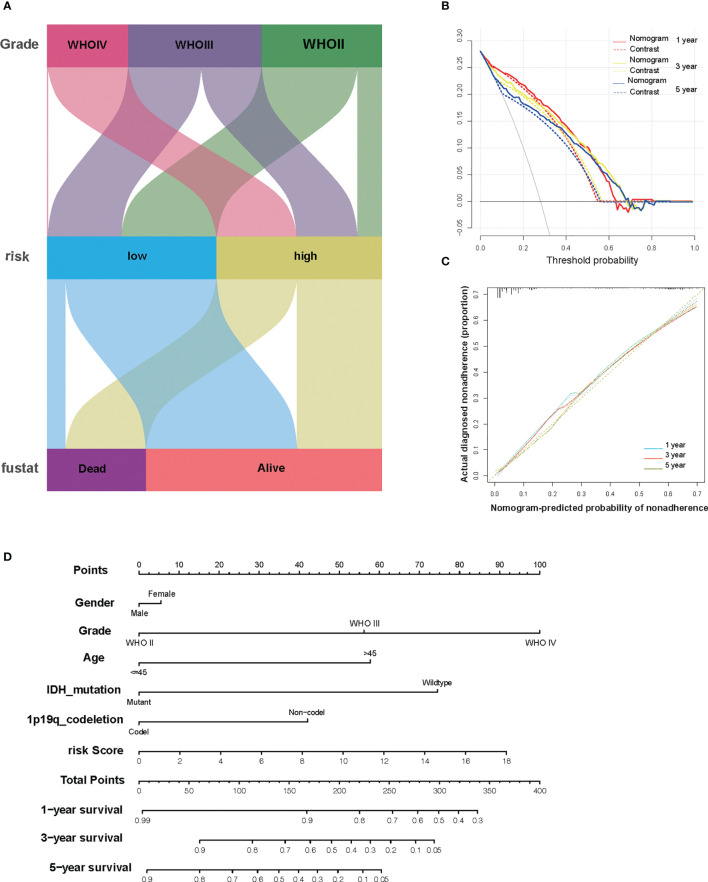
The combination of risk signature and clinicopathological features improves risk stratification and survival prediction. **(A)** A decision tree was constructed to improve risk stratification. **(B)** Decision curve analysis of the nomogram and contrast system for predicting the 1-, 3-, and 5-year survival rate of patients. **(C)** The calibration analysis indicated a high accuracy of survival prediction. **(D)** A nomogram was constructed to quantify risk assessment for individual patients.

To better enhance the risk stratification of prognosis, we constructed a decision tree through patients with different grades of glioma from TCGA. As a result, the difference in overall survival was observed in subgroups with different risk scores (**Figure 6A**). Developing individualized treatment for individual glioma patients is necessary. Consistently assessing the potential risk and prognosis for individual glioma patients is also important. Consequently, we built a nomogram with risk score as well as clinical pathology features, including IDH mutation and 1p19q (**Figure 6D**). Besides this, the calibration analysis was performed to elevate the accuracy of our nomogram. The results showed that the prediction line of the nomogram was extremely close to the ideal performance (45° dotted line) (**Figures 6B, C** **)**.

### The Differences in Immunocyte Infiltration Degree and Enrichment Plots of Immune-Related Gene Sets From Gene Set Enrichment Analysis Between High- and Low-Risk TCGA Cohorts

**Figure 7:**
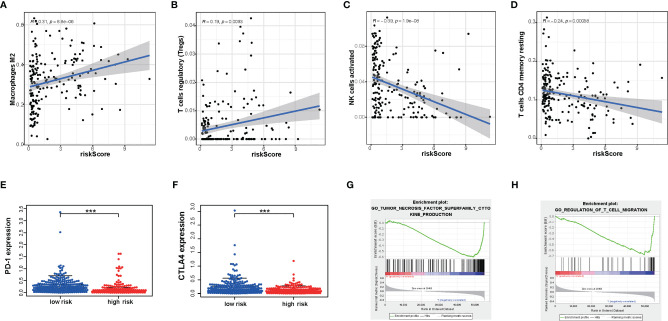
The differences in immunocyte infiltration degree and enrichment plots of immune-related gene sets from Gene Set Enrichment Analysis (GSEA) between the high- and low-risk TCGA cohorts. **(A**–**D)** The correlation with immunocyte infiltration was performed by using Pearson correlation analysis. M2; Tregs; NK cells; CD4+T cells. **(E**, **F)** Correlation with immune-checkpoint expression. PD-1; CTLA4. **(G**, **H)** GSEA analysis revealing immune-related biological processes correlated with the signature. ***P < 0.001.

Next, to explore whether our risk score partly assessed the immune status of the tumor microenvironment, the relationship of amino acid metabolism- and immune-related gene signature with the immunocyte infiltration degree was explored in gliomas. Interestingly, our results indicated that M2 (Cor = 0.31; *p* = 8.8e−6) and Tregs (Cor = 0.169; *p* = 0.0093) were obviously positively related to risk score (**Figures 7A, B** **)**. Furthermore, NK cells (Cor = -0.39; p =1.9e−08) and CD4+ T cells (Cor = -0.24; p = 0.00058) (**Figures 7C, D** **)** showed a negative correlation with the risk score.

Immunotherapy is increasingly becoming an important part of tumor therapy and can significantly improve the prognosis of cancer patients in a variety of solid tumors (25). Hence, we detected the expression of immune checkpoints in subgroups with a high or low risk score. According to our gene model, the expression level of PD- L1, PD-1, and CTLA-4 was lower in glioma patients with a high risk score (*P* < 0.05) (**Figures 7E, F** **)**. This result showed that the high-risk-score group may be less sensitive to immunotherapy. Furthermore, glioma with a high risk score was obviously enriched in the downregulation of the effect immunity pathway. We found that high risk score had a negative relationship with T cell migration (**Figure 7G**). It is well known that enhancing T cell infiltration in glioma may increase the response rates to immunotherapy and increase survival. Consistently, high risk score had a negative relationship with tumor necrosis factor function (**Figure 7H**). These results indicated that risk score could predict an immunosuppressive micro-environment.

### Identification of Hub Genes From Risk Signature as Biomarkers in Glioma

**Figure 8:**
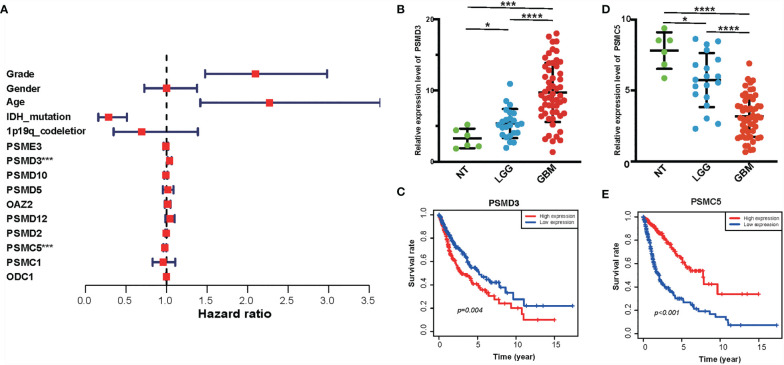
Identification of hub genes of amino metabolism-related risk signature. **(A)** Forest plot of the multivariable Cox regression analysis of the effect of 10 hub genes and clinicopathological variables on the overall survival (OS) of glioma patients. **(B)** The relative expression level of PSMD3 in normal, low-grade glioma (LGG), and glioblastoma (GBM) tissues according to the rt-PCR results. **(C)** The OS survival analysis of PSMD3 in TCGA glioma. **(D)** The relative expression level of PSMC5 in normal, LGG, and GBM tissues according to the rt-PCR results. **(E)** The OS survival analysis of PSMC5 in TCGA glioma. *P < 0.05, ***P < 0.001, ****P < 0.0001.

The PPI network among the overlapped genes was established by using the STRING database and performing GO and KEGG (**Supplementary Figure S3**). MCC algorithm of CytoHubba plugin was used to select hub genes of the PPI network, and the hub genes are listed in **Supplementary Table S1**. Basing on the MCC scores, we selected the top 10 highest-scored genes from hub genes, including ODC1, OAZ2, PSMD2, PSMD12, PSMC1, PSMC5, PSMD3, PSME3, PSMD10, and PSMD5. The expression levels of these genes were verified according to the TCGA database. Kaplan–Meier plotter and the expression level of the top 10 genes were performed as shown in **Supplementary Figure S4** and **Supplementary Figure S5**. Then, we performed a multivariate Cox analysis to evaluate the prognostic value of these genes in gliomas (**Figure 8A** and **Supplementary Table S2**). In addition, we performed RT-PCR in our clinical samples which include six normal brain tissues, 24 WHO grade II, and 55 GBM samples. Consistently, we found that the expression level of PSMD3 was positive with the grade of glioma. Inversely, the expression level of PSMC5 was negative with the grade of glioma (**Figures 8B, D** **)**. Moreover, the results of the Kaplan–Meier analysis indicated that PSMD3 was significantly associated with worse overall survival of the glioma patients (*P* < 0.05) (**Figure 8C**). Conversely, PSMC5 was significantly associated with better overall survival of the glioma patients (*P* < 0.05) (**Figure 8E**). All the results confirmed that PSMD3 and PSMC5 can be identified as potential biomarkers in glioma patients.

## Discussion

The reprogramming of amino acid metabolism in gliomas has been reported to contribute to the malignancy biological process of glioma, including proliferation, migration, and so on. A previous study has constructed an amino acid-related risk signature for gliomas, which could predict the survival and clinical features of patients (26). However, more and more studies revealed that amino acid metabolism is not only caused by oncogene alterations but also changed the surrounding tumor microenvironment (27).

In our study, we focused on the amino acid metabolism and immune status which was explored by ssGSEA and ESTIMATE and confirmed the differential expression of genes in gliomas. Then, WGCNA was performed to identify amino acid metabolism and immuno-related gene modules based on the data from TCGA, and an amino acid metabolism and immune signature was constructed by LASSO Cox regression model. Subsequently, the prognostic value of the gene signature was validated in CGGA cohorts. Our risk score system could distinguish high-risk patients, indicating that it can act as a confidential risk factor in the complex subgroups of patients. In addition, a decision tree has been constructed to enhance risk stratification on the basis of WHO grade and risk score, which showed that risk score could act as a major determinant. Moreover, the generation of the nomogram was used to quantify risk assessment and survival probability, which could show higher accuracy and discrimination in survival prediction compared with the traditional characteristics.

Furthermore, our risk score could also provide valuable information on immune cell infiltration in a tumor microenvironment and reflect the effects of immunotherapy. In the high-risk-score subgroup, the infiltration of immunosuppressive cells like Tregs is higher than in the low-risk-score subgroup. Conversely, effect immune cells are decreased in the high-risk-score subgroup. Consistently, a recent study has shown that amino acid metabolism can regulate immune cells in cancer (3, 28, 29). Our gene model might provide a clue of how the microenvironment was influenced by amino acid metabolism. In addition, immune checkpoint therapy has shown a great potential for treatment in diverse solid tumors (30). However, the therapeutic efficacy has not lived up to expectations in gliomas, and the specific mechanisms for the problem still need more research. Different genomic subtypes or molecular profiles are the main challenges in the response to PD-1/PD-L1 checkpoint blockades (31). In addition, the amino acid derivatives could promote the immunosuppressive microenvironment and even affect the expression of immune checkpoints in glioma (32, 33). Interestingly, the expression of PD-L1 and CTLA-4 in the high-risk group was significantly lower than in the low-risk group in our study. These results might indicate better efficacy and greater sensitivity of anti-PD1 therapy in low-risk glioma patients.

In our study, we identified two biomarkers by estimating amino acid and immune status in gliomas on the basis of the expression of mRNA. PSMD3, also known as P58 or RPN3, is one of the members in the proteasome subunit S3 family, which acts as the non-ATPase subunit of the 19S regulator lid (34). PSMD3 is widely expressed in most tissues and defined as an oncogene in various cancers. WBC and neutrophil counts are related to the expression of PSMD3 (35). Additionally, PSMD3 is also related to the glucose-related features of carbohydrates and fatty acids from the diet (36, 37). Besides this, the higher level of PSMD3 mRNA predicts a worse prognosis of acute myeloid leukemia patients, and PSMD3 promotes the progression of chronic myeloid leukemia by stabilizing NF-kB (38, 39). Consistently, PSMD3 is upregulated in breast cancer compared with normal tissue, and patients with a higher expression level of PSMD3 are related with worse survival. PSMD3 is strongly associated with the expression of HER2, which can stabilize HER2 from degradation (40). PSMC5 is defined as a 19S regulatory component, and it could identify and transform ubiquitin-labeled proteins into the form of degradation which can be mediated by 20S complex (41). Interestingly, PSMC5 directly regulates transcription, for instance, it can influence the activity class II trans-activator to regulate the transcription of MHC class II (42). Besides this, it can also recruit p53 to the promoter of p21 to upregulate its expression, which can decrease the DNA damage mediated by ultraviolet (43), yet the specific functions of both biomarkers in gliomas remain unclear. More research are needed for further study.

Finally, several limitations of our work should be mentioned, namely: (1) although two biomarkers have been identified, the potential function of these genes still remains unclear and should be explored in a future study, and (2) tumor heterogeneity is one of the most important features in gliomas (44), which also means different microenvironment features exist among diverse tumor sites, yet all of the data and information are collected from public databases, which makes it impossible to detect the immune status in the same or diverse tumor regions. As a result, this gene signature should be better validated in well-designed, multicenter, prospective studies.

## Conclusions

In summary, the construction and validation of 12 amino acid metabolism- and immune-related genes have been defined as a prognostic signature. This prognostic signature can predict the prognosis of patients and help to select individualized therapeutic strategy in clinical practice, which provides a comprehensive perspective for clarifying the underlying mechanisms that determine the prognosis for glioma. In addition, our risk score model is associated with the immune status of glioma patients, which may imply the potential effect of immuno-therapy. Besides this, we also have identified PSMC5 and PSMD3 as new biomarkers in glioma.

## Data Availability Statement

The datasets presented in this study can be found in online repositories. The names of the repository/repositories and accession number(s) can be found in the article/**Supplementary Material**.

## Ethics Statement

The studies involving human participants were reviewed and approved by the Institutional Ethics Committee of the Faculty of Medicine at Renmin Hospital of Wuhan University. The patients/participants provided their written informed consent to participate in this study.

## Author Contributions

QC and FY designed the research. YX, LY, and RG carried out the work. PH, QS, and ST analyzed the data and wrote the paper. All authors contributed to the article and approved the submitted version.

## Funding

This work was supported by the National Natural Science Foundation of China (no. 82072764).

## Conflict of Interest

The authors declare that the research was conducted in the absence of any commercial or financial relationships that could be construed as a potential conflict of interest.

## Publisher’s Note

All claims expressed in this article are solely those of the authors and do not necessarily represent those of their affiliated organizations, or those of the publisher, the editors and the reviewers. Any product that may be evaluated in this article, or claim that may be made by its manufacturer, is not guaranteed or endorsed by the publisher.

## Supplementary Material

Supplementary Figure 1 Identification of differentially expressed amino metabolism-related genes among glioma and normal brain tissues. **(A)** The Venn plot shows the intersecting genes between differentially expressed genes among glioma and amino metabolism-related genes. **(B)** The Gene Ontology and **(C)** Kyoto Encyclopedia of Genes and Genomes analysis of the intersecting genes. **(D)** The protein–protein intersection analysis of the intersecting genes.

Supplementary Figure 2 Identification of immune subtypes in The Cancer Genome Atlas (TCGA) glioma by single-sample gene set enrichment analysis. **(A)** Hierarchical clustering of glioma yields two subtypes (immune-high group and immune-low group) in the TCGA database. **(B)** Different immune status of the two groups. **(C)** A comparison of the abundance of tumor-infiltrating immune cells between immune-high and immune-low groups is shown. A comparison of **(D)** immune score, **(E)** stromal score, **(F)** estimate score, and **(G)** tumor purity among immune-high and immune-low groups is shown. **(H)** Survival analysis of overall survival between patients in the immune-low and immune-high groups. **P* < 0.05, ***P* < 0.01, ****P* < 0.001.

Supplementary Figure 3 Screened out top 10 hub genes of differentially expressed amino metabolism-related genes. **(A)** The colors of the hub genes were ranked by Maximal Clique Centrality (MCC) value, calculated through Cytoscape plug-in Cytohubba; the depth of the color represents the MCC value. **(B)** Gene Ontology and Kyoto Encyclopedia of Genes and Genomes analysis of the amino metabolism-related genes.

Supplementary Figure 4 The expression level of these 10 hub genes in normal, low-grade glioma (LGG), and glioblastoma (GBM) tissues in the training set. The mRNA expression level of **(A)** PSME3, **(B)** PSMD3, **(C)** PSMD10, **(D)** PSMD5, **(E)** OAZ2, **(F)** PSMD12, **(G)** PSMD2, **(H)** PSMC5, **(I)** PSMC1, and **(J)** ODC1 in normal, LGG, and GBM tissues in the training set.

Supplementary Figure 5 The prognostic value of these 10 hub genes in glioma. Kaplan–Meier survival analysis of **(A)** PSME3, **(B)** PSMD3, **(C)** PSMD10, **(D)** PSMD5, **(E)** OAZ2, **(F)** PSMD12, **(G)** PSMD2, **(H)** PSMC5, **(I)** PSMC1, and **(J)** ODC1 was performed for overall survival in The Cancer Genome Atlas glioma.

## Abbreviations

PD-L1, programmed cell death 1 ligand; IDO, indolamine 2,3-dioxygenase; TGF-β, transforming growth factor β; TCGA, The Cancer Genome Atlas; CGGA, Chinese Glioma Genome Atlas; ssGSEA, single-sample Gene Set Enrichment Analysis; WGCNA, weighted gene co-expression network analysis; LASSO, least absolute shrinkage and selection operator; ICI, immune checkpoint inhibitor; PPI, protein–protein interaction; WBC, white blood cell; ICB, immune checkpoint blockade; OS, overall survival; IDO, indoleamine-2,3-dioxygenase; TDO, tryptophan-2,3-dioxygenase.

## References

[1] Dang CV. Links Between Metabolism and Cancer. Genes Dev (2012) 26:877–90. doi: 10.1101/gad.189365.112 PMC3347786 22549953

[2] Sivanand S Vander Heiden MG. Emerging Roles for Branched-Chain Amino Acid Metabolism in Cancer. Cancer Cell (2020) 37:147–56. doi: 10.1016/j.ccell.2019.12.011 PMC7082774 32049045

[3] Lemos H Huang L Prendergast GC Mellor AL. Immune Control by Amino Acid Catabolism During Tumorigenesis and Therapy. Nat Rev Cancer (2019) 19:162–75. doi: 10.1038/s41568-019-0106-z 30696923

[4] Guccione E Richard S. The Regulation, Functions and Clinical Relevance of Arginine Methylation. Nat Rev Mol Cell Biol (2019) 20:642–57. doi: 10.1038/s41580-019-0155-x 31350521

[5] Greene LI Bruno TC Christenson JL D’Alessandro A Culp-Hill R Torkko K. A Role for Tryptophan-2,3-Dioxygenase in CD8 T-Cell Suppression and Evidence of Tryptophan Catabolism in Breast Cancer Patient Plasma. Mol Cancer Res (2019) 17:131–9. doi: 10.1158/1541-7786.MCR-18-0362 PMC6318037 30143553

[6] Opitz CA Litzenburger UM Sahm F Ott M Tritschler I Trump S. An Endogenous Tumour-Promoting Ligand of the Human Aryl Hydrocarbon Receptor. Nature (2011) 478:197–203. doi: 10.1038/nature10491 21976023

[7] Fallarino F Grohmann U Hwang KW Orabona C Vacca C Bianchi R. Modulation of Tryptophan Catabolism by Regulatory T Cells. Nat Immunol (2003) 4:1206–12. doi: 10.1038/ni1003 14578884

[8] Commisso C Davidson SM Soydaner-Azeloglu RG Parker SJ Kamphorst JJ Hackett S. Macropinocytosis of Protein Is an Amino Acid Supply Route in Ras-Transformed Cells. Nature (2013) 497:633–7. doi: 10.1038/nature12138 PMC3810415 23665962

[9] Ostrom QT Patil N Cioffi G Waite K Kruchko C Barnholtz-Sloan JS. CBTRUS Statistical Report: Primary Brain and Other Central Nervous System Tumors Diagnosed in the United States in 2013-2017. Neuro Oncol (2020) 22:iv1–iv96. doi: 10.1093/neuonc/noaa200 33123732 PMC7596247

[10] Tan AC Ashley DM López GY Malinzak M Friedman HS Khasraw M. Management of Glioblastoma: State of the Art and Future Directions. CA Cancer J Clin (2020) 70:299–312. doi: 10.3322/caac.21613 32478924

[11] Lim M Xia Y Bettegowda C Weller M. Current State of Immunotherapy for Glioblastoma. Nat Rev Clin Oncol (2018) 15:422–42. doi: 10.1038/s41571-018-0003-5 29643471

[12] Wainwright DA Balyasnikova IV Chang AL Ahmed AU Moon K-S Auffinger B. IDO Expression in Brain Tumors Increases the Recruitment of Regulatory T Cells and Negatively Impacts Survival. Clin Cancer Res (2012) 18:6110–21. doi: 10.1158/1078-0432.CCR-12-2130 PMC3500434 22932670

[13] Bloch O Crane CA Kaur R Safaee M Rutkowski MJ Parsa AT. Gliomas Promote Immunosuppression Through Induction of B7-H1 Expression in Tumor-Associated Macrophages. Clin Cancer Res (2013) 19:3165–75. doi: 10.1158/1078-0432.CCR-12-3314 PMC3742575 23613317

[14] Colombo MP Piconese S. Regulatory-T-Cell Inhibition *Versus* Depletion: The Right Choice in Cancer Immunotherapy. Nat Rev Cancer (2007) 7:880–7. doi: 10.1038/nrc2250 17957190

[15] Liberzon A Birger C Thorvaldsdóttir H Ghandi M Mesirov JP Tamayo P. The Molecular Signatures Database (Msigdb) Hallmark Gene Set Collection. Cell Syst (2015) 1:417–25. doi: 10.1016/j.cels.2015.12.004 PMC4707969 26771021

[16] Miranda A Hamilton PT Zhang AW Pattnaik S Becht E Mezheyeuski A. Cancer Stemness, Intratumoral Heterogeneity, and Immune Response Across Cancers. Proc Natl Acad Sci USA (2019) 116:9020–9. doi: 10.1073/pnas.1818210116 PMC6500180 30996127

[17] Yoshihara K Shahmoradgoli M Martínez E Vegesna R Kim H Torres-Garcia W. Inferring Tumour Purity and Stromal and Immune Cell Admixture From Expression Data. Nat Commun (2013) 4:2612. doi: 10.1038/ncomms3612 24113773 PMC3826632

[18] Tibshirani R. The Lasso Method for Variable Selection in the Cox Model. Stat Med (1997) 16:385–95. doi: 10.1002/(sici)1097-0258(19970228)16:4<385::aid-sim380>3.0.co;2-3 9044528

[19] Heagerty PJ Lumley T Pepe MS. Time-Dependent ROC Curves for Censored Survival Data and a Diagnostic Marker. Biometrics (2000) 56:337–44. doi: 10.1111/j.0006-341x.2000.00337.x 10877287

[20] Newman AM Liu CL Green MR Gentles AJ Feng W Xu Y. Robust Enumeration of Cell Subsets From Tissue Expression Profiles. Nat Methods (2015) 12:453–7. doi: 10.1038/nmeth.3337 PMC4739640 25822800

[21] Gene Ontology Consortium. The Gene Ontology (GO) Project in 2006. Nucleic Acids Res (2006) 34:D322–326. doi: 10.1093/nar/gkj021 PMC1347384 16381878

[22] Shannon P Markiel A Ozier O Baliga NS Wang JT Ramage D. Cytoscape: A Software Environment for Integrated Models of Biomolecular Interaction Networks. Genome Res (2003) 13:2498–504. doi: 10.1101/gr.1239303 PMC403769 14597658

[23] Wu Z Liu Y Xu J Xie J Zhang S Huang L. A Ventilator-Associated Pneumonia Prediction Model in Patients With Acute Respiratory Distress Syndrome. Clin Infect Dis (2020) 71:S400–8. doi: 10.1093/cid/ciaa1518 33367575

[24] Lapointe S Perry A Butowski NA. Primary Brain Tumours in Adults. Lancet (2018) 392:432–46. doi: 10.1016/S0140-6736(18)30990-5 30060998

[25] Jackson CM Choi J Lim M. Mechanisms of Immunotherapy Resistance: Lessons From Glioblastoma. Nat Immunol (2019) 20:1100–9. doi: 10.1038/s41590-019-0433-y 31358997

[26] Liu Y-Q Chai R-C Wang Y-Z Wang Z Liu X Wu F. Amino Acid Metabolism-Related Gene Expression-Based Risk Signature can Better Predict Overall Survival for Glioma. Cancer Sci (2019) 110:321–33. doi: 10.1111/cas.13878 PMC6317920 30431206

[27] Kesarwani P Prabhu A Kant S Chinnaiyan P. Metabolic Remodeling Contributes Towards an Immune-Suppressive Phenotype in Glioblastoma. Cancer Immunol Immunother (2019) 68:1107–20. doi: 10.1007/s00262-019-02347-3 PMC6586493 31119318

[28] Li Z Zhang H. Reprogramming of Glucose, Fatty Acid and Amino Acid Metabolism for Cancer Progression. Cell Mol Life Sci (2016) 73:377–92. doi: 10.1007/s00018-015-2070-4 PMC11108301 26499846

[29] Timosenko E Hadjinicolaou AV Cerundolo V. Modulation of Cancer-Specific Immune Responses by Amino Acid Degrading Enzymes. Immunotherapy (2017) 9:83–97. doi: 10.2217/imt-2016-0118 28000524

[30] Li B Chan HL Chen P. Immune Checkpoint Inhibitors: Basics and Challenges. Curr Med Chem (2019) 26:3009–25. doi: 10.2174/0929867324666170804143706 28782469

[31] Cloughesy TF Mochizuki AY Orpilla JR Hugo W Lee AH Davidson TB. Neoadjuvant Anti-PD-1 Immunotherapy Promotes a Survival Benefit With Intratumoral and Systemic Immune Responses in Recurrent Glioblastoma. Nat Med (2019) 25:477–86. doi: 10.1038/s41591-018-0337-7 PMC6408961 30742122

[32] Adams S Teo C McDonald KL Zinger A Bustamante S Lim CK. Involvement of the Kynurenine Pathway in Human Glioma Pathophysiology. PloS One (2014) 9:e112945. doi: 10.1371/journal.pone.0112945 25415278 PMC4240539

[33] Liu Y Liang X Dong W Fang Y Lv J Zhang T. Tumor-Repopulating Cells Induce PD-1 Expression in CD8+ T Cells by Transferring Kynurenine and Ahr Activation. Cancer Cell (2018) 33:480–94.e7. doi: 10.1016/j.ccell.2018.02.005 29533786

[34] Rubio AJ Bencomo-Alvarez AE Young JE Velazquez VV Lara JJ Gonzalez MA. 26S Proteasome Non-Atpase Regulatory Subunits 1 (PSMD1) and 3 (PSMD3) as Putative Targets for Cancer Prognosis and Therapy. Cells (2021) 10:2390. doi: 10.3390/cells10092390 34572038 PMC8472613

[35] Okada Y Kamatani Y Takahashi A Matsuda K Hosono N Ohmiya H. Common Variations in PSMD3-CSF3 and PLCB4 Are Associated With Neutrophil Count. Hum Mol Genet (2010) 19:2079–85. doi: 10.1093/hmg/ddq080 20172861

[36] Zheng J-S Arnett DK Parnell LD Lee Y-C Ma Y Smith CE. Genetic Variants at PSMD3 Interact With Dietary Fat and Carbohydrate to Modulate Insulin Resistance. J Nutr (2013) 143:354–61. doi: 10.3945/jn.112.168401 PMC3713024 23303871

[37] Iio E Matsuura K Nishida N Maekawa S Enomoto N Nakagawa M. Genome-Wide Association Study Identifies a PSMD3 Variant Associated With Neutropenia in Interferon-Based Therapy for Chronic Hepatitis C. Hum Genet (2015) 134:279–89. doi: 10.1007/s00439-014-1520-7 25515861

[38] Dai Y-J Hu F He S-Y Wang Y-Y. Epigenetic Landscape Analysis of Lncrnas in Acute Myeloid Leukemia With DNMT3A Mutations. Ann Transl Med (2020) 8:318. doi: 10.21037/atm.2020.02.143 32355762 PMC7186694

[39] Bencomo-Alvarez AE Rubio AJ Olivas IM Gonzalez MA Ellwood R Fiol CR. Proteasome 26S Subunit, Non-Atpases 1 (PSMD1) and 3 (PSMD3), Play an Oncogenic Role in Chronic Myeloid Leukemia by Stabilizing Nuclear Factor-Kappa B. Oncogene (2021) 40:2697–710. doi: 10.1038/s41388-021-01732-6 PMC7952820 33712704

[40] Fararjeh AS Chen L-C Ho Y-S Cheng T-C Liu Y-R Chang H-L. Proteasome 26S Subunit, Non-Atpase 3 (PSMD3) Regulates Breast Cancer by Stabilizing HER2 From Degradation. Cancers (Basel) (2019) 11:E527. doi: 10.3390/cancers11040527 31013812 PMC6549480

[41] Yim J-H Yun HS Lee S-J Baek J-H Lee C-W Song J-Y. Radiosensitizing Effect of PSMC5, A 19S Proteasome Atpase, in H460 Lung Cancer Cells. Biochem Biophys Res Commun (2016) 469:94–100. doi: 10.1016/j.bbrc.2015.11.077 26592665

[42] Bhat KP Turner JD Myers SE Cape AD Ting JP-Y Greer SF. The 19S Proteasome Atpase Sug1 Plays a Critical Role in Regulating MHC Class II Transcription. Mol Immunol (2008) 45:2214–24. doi: 10.1016/j.molimm.2007.12.001 18215421

[43] Zhu Q Wani G Yao J Patnaik S Wang Q-E El-Mahdy MA. The Ubiquitin-Proteasome System Regulates P53-Mediated Transcription at P21waf1 Promoter. Oncogene (2007) 26:4199–208. doi: 10.1038/sj.onc.1210191 17224908

[44] Puchalski RB Shah N Miller J Dalley R Nomura SR Yoon J-G. An Anatomic Transcriptional Atlas of Human Glioblastoma. Science (2018) 360:660–3. doi: 10.1126/science.aaf2666 PMC6414061 29748285

