# Supplementary figures and images for: Development and Verification of the Amino Metabolism-Related and Immune-Associated Prognosis Signature in Gliomas
Source: Front Oncol. 2021 Nov 5;11:774332. doi: 10.3389/fonc.2021.774332 (PMC8602207; doi:10.3389/fonc.2021.774332)

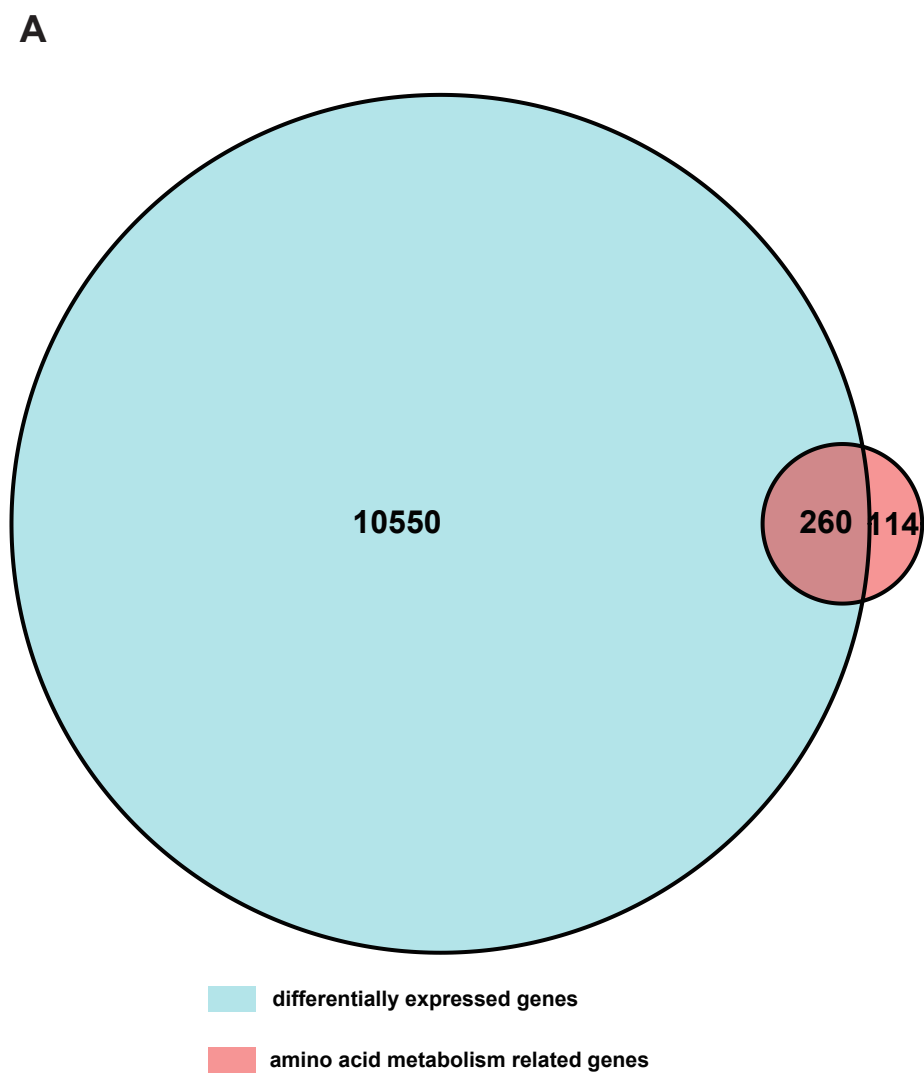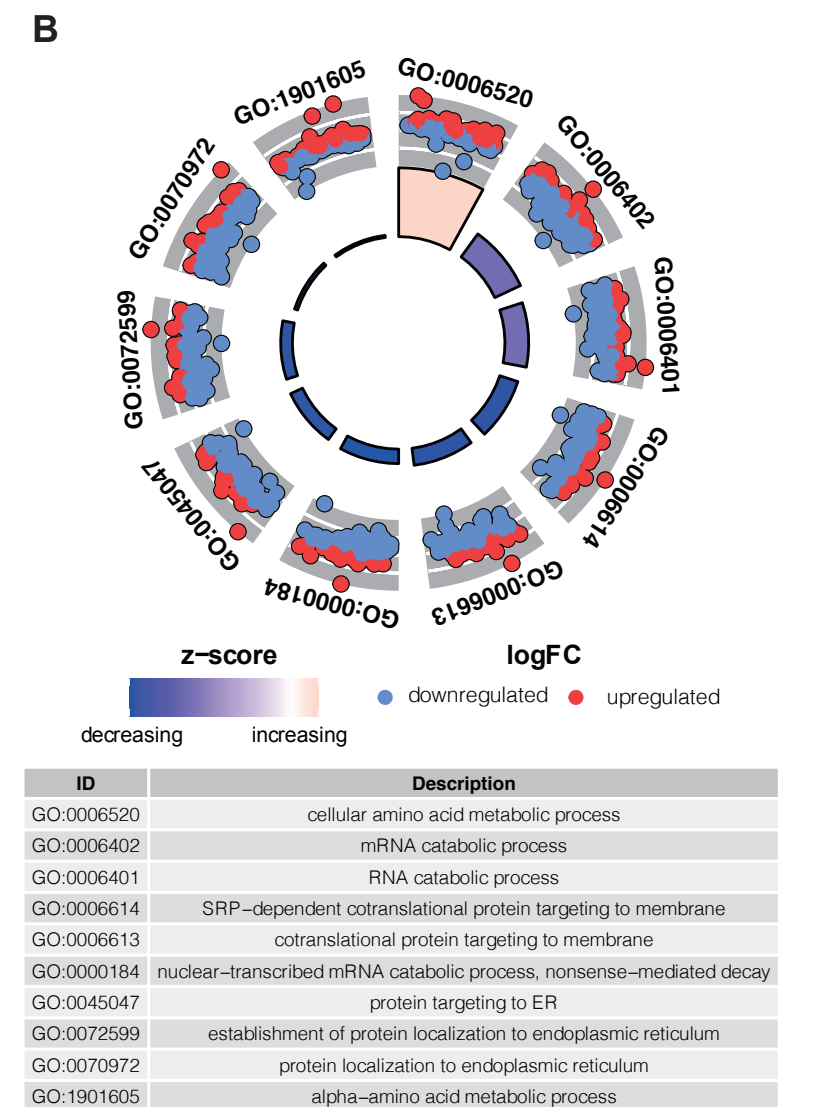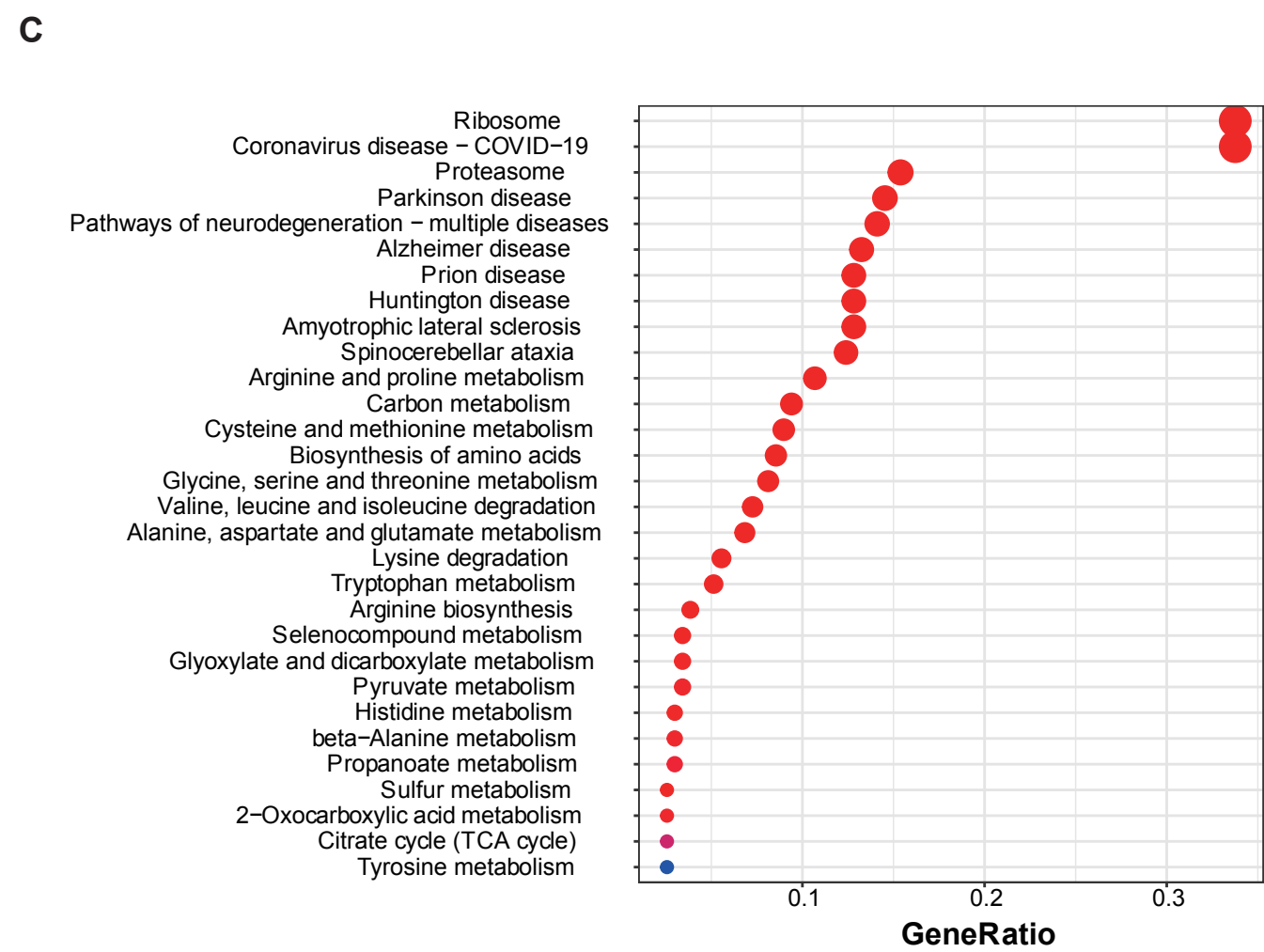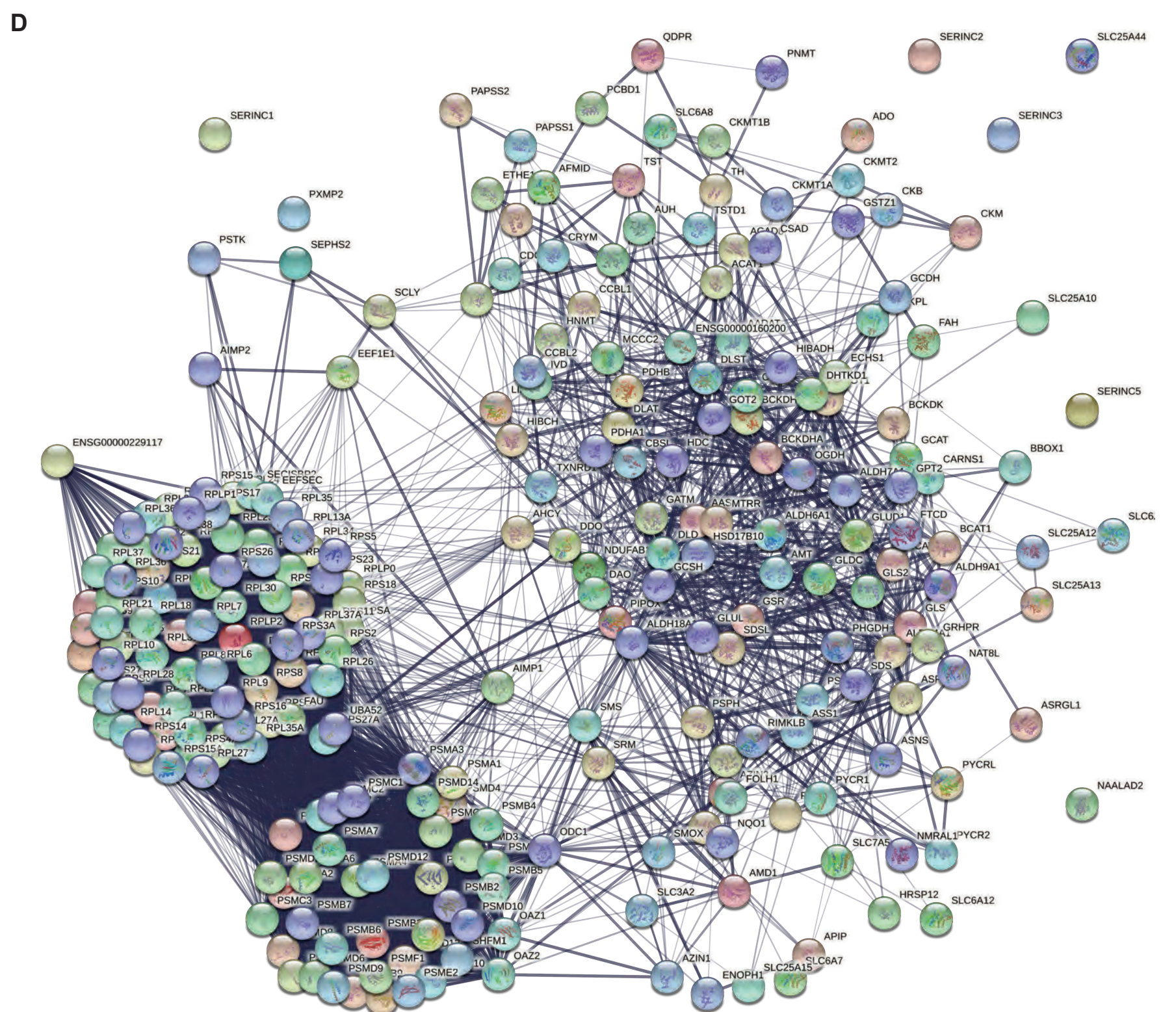

Supplement: Supplementary Figure 1 — Identification of differentially expressed amino metabolism-related genes among glioma and normal brain tissues. (A) The Venn plot shows the intersecting genes between differentially expressed genes among glioma and amino metabolism-related genes. (B) The Gene Ontology and (C) Kyoto Encyclopedia of Genes and Genomes analysis of the intersecting genes. (D) The protein–protein intersection analysis of the intersecting genes. [file DataSheet_1.pdf]

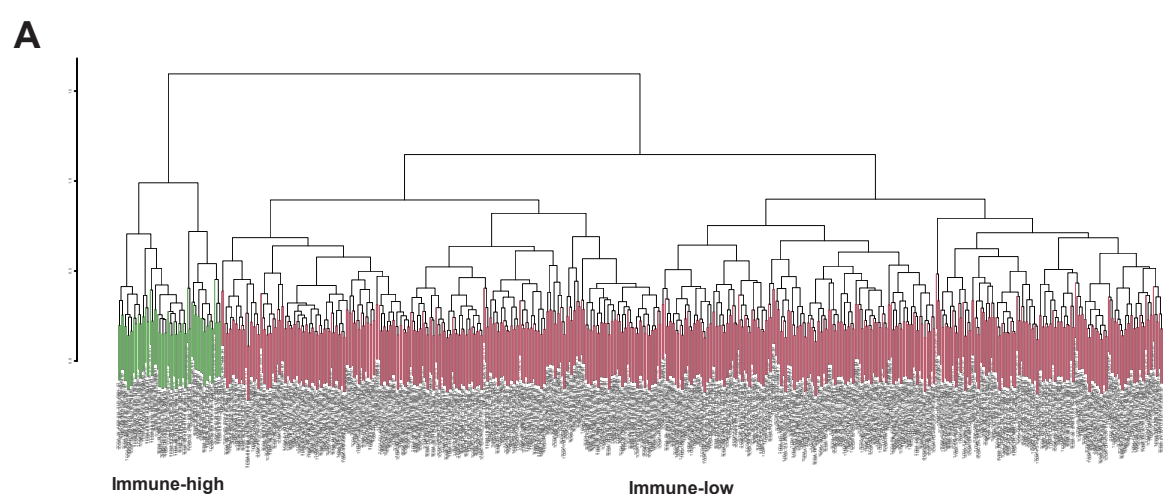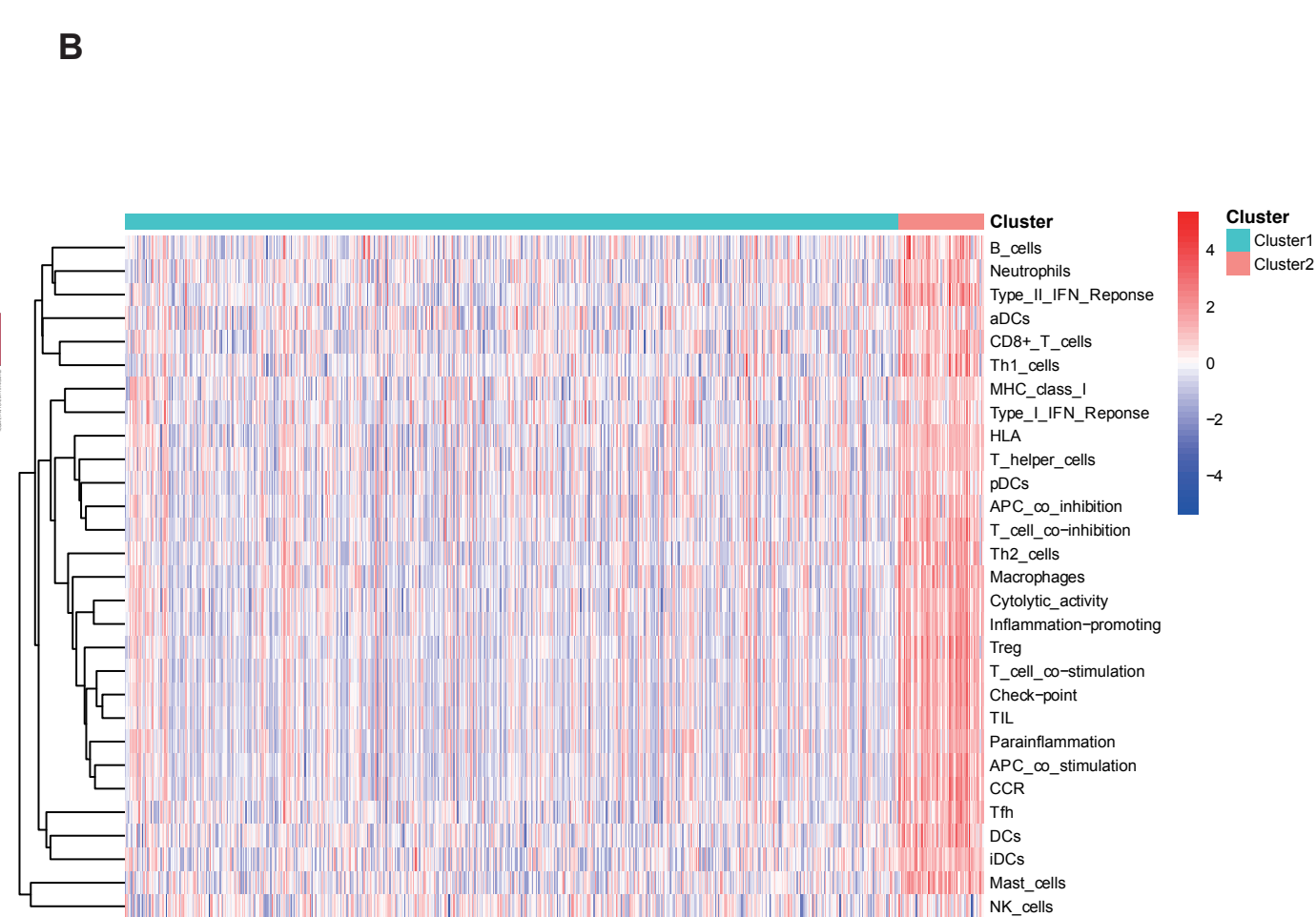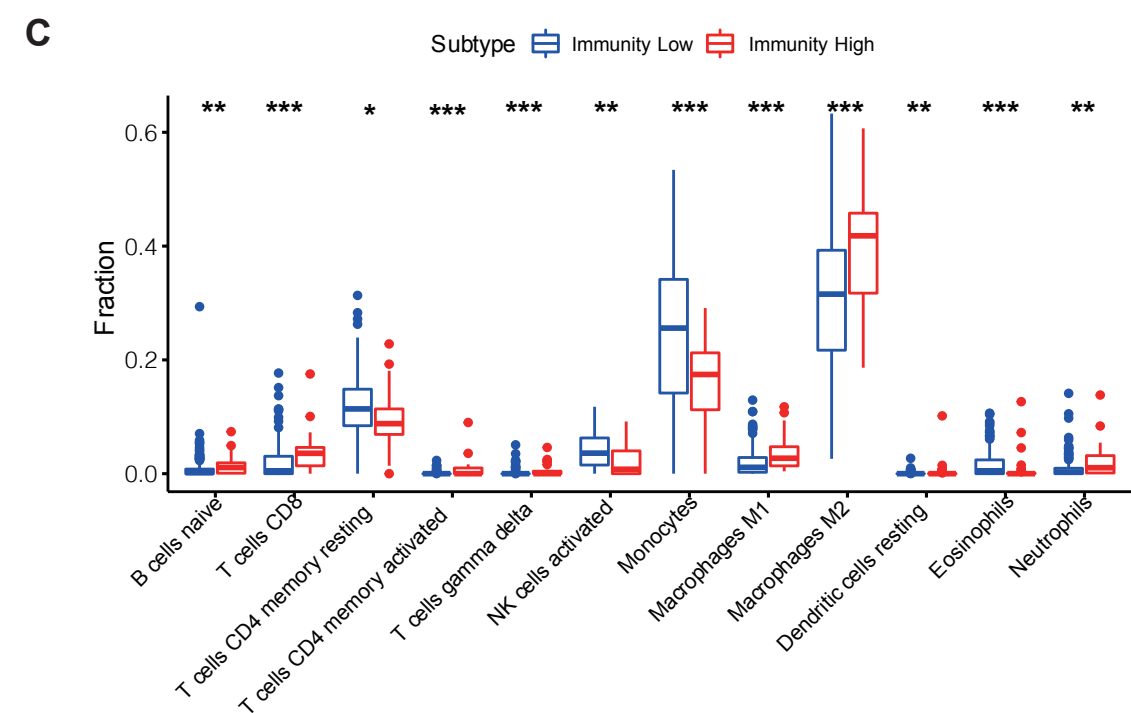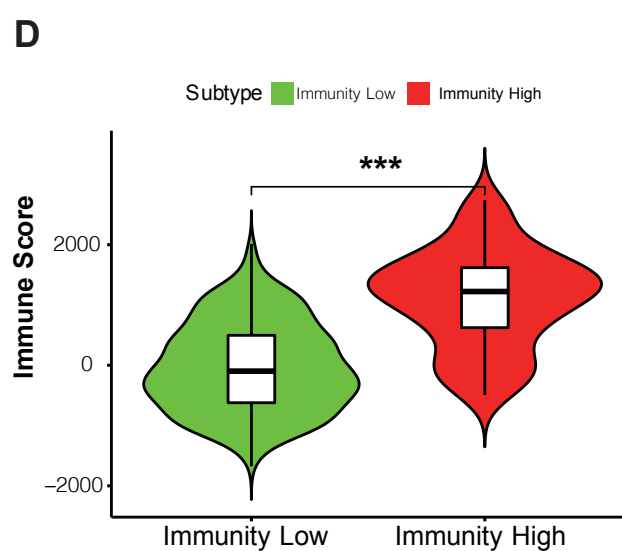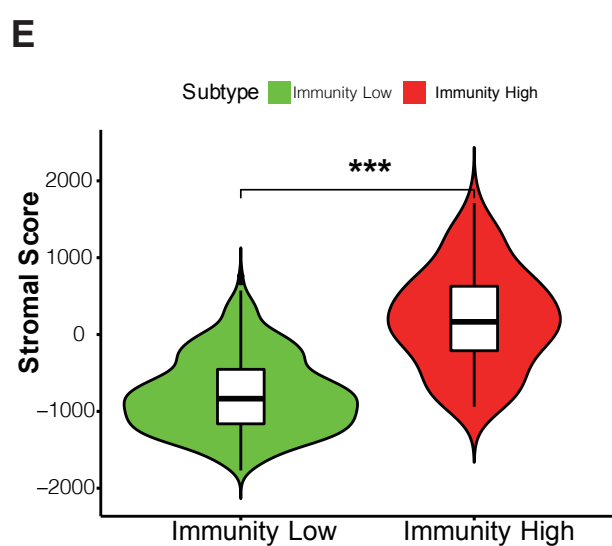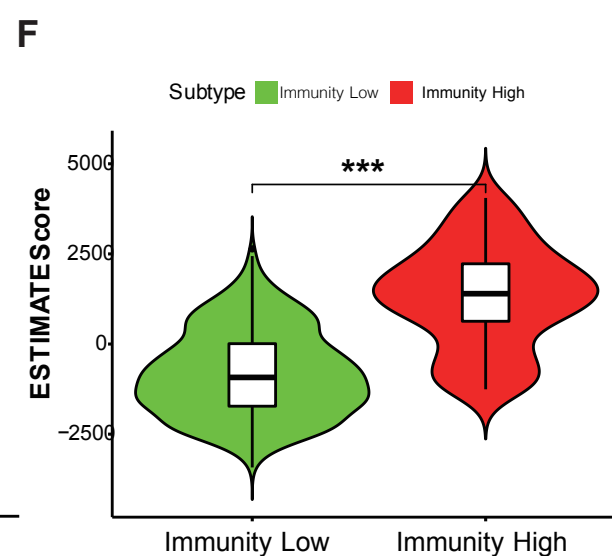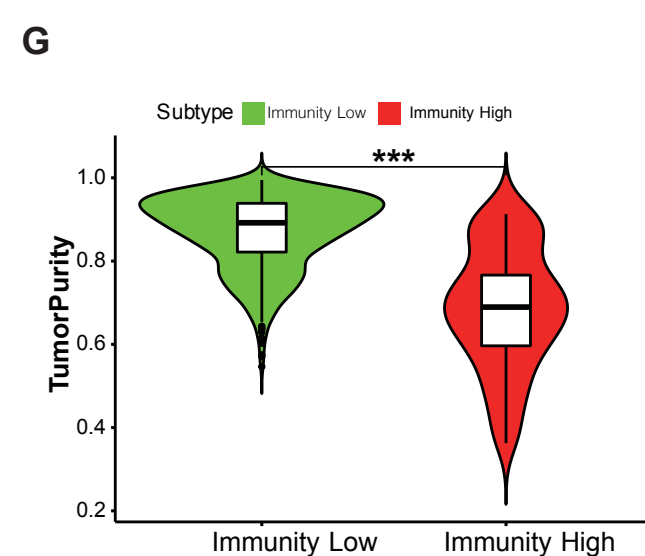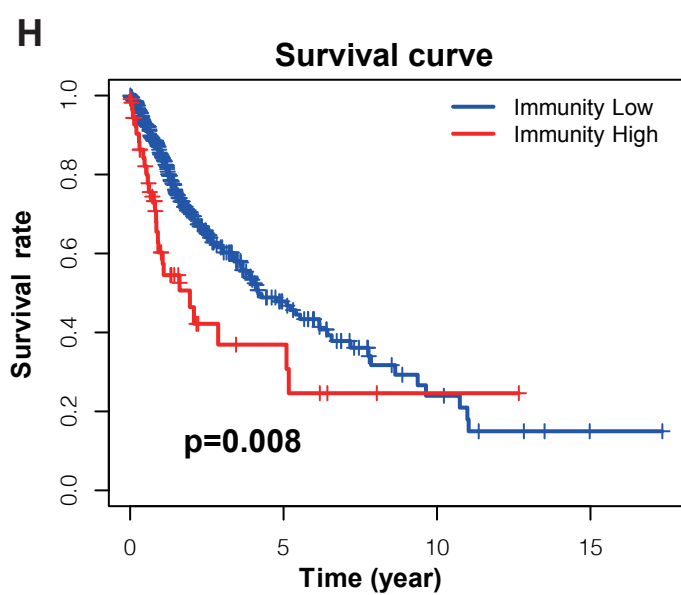

Supplement: Supplementary Figure 2 — Identification of immune subtypes in The Cancer Genome Atlas (TCGA) glioma by single-sample gene set enrichment analysis. (A) Hierarchical clustering of glioma yields two subtypes (immune-high group and immune-low group) in the TCGA database. (B) Different immune status of the two groups. (C) A comparison of the abundance of tumor-infiltrating immune cells between immune-high and immune-low groups is shown. A comparison of (D) immune score, (E) stromal score, (F) estimate score, and (G) tumor purity among immune-high and immune-low groups is shown. (H) Survival analysis of overall survival between patients in the immune-low and immune-high groups. *P < 0.05, **P < 0.01, ***P < 0.001. [file DataSheet_2.pdf]

A

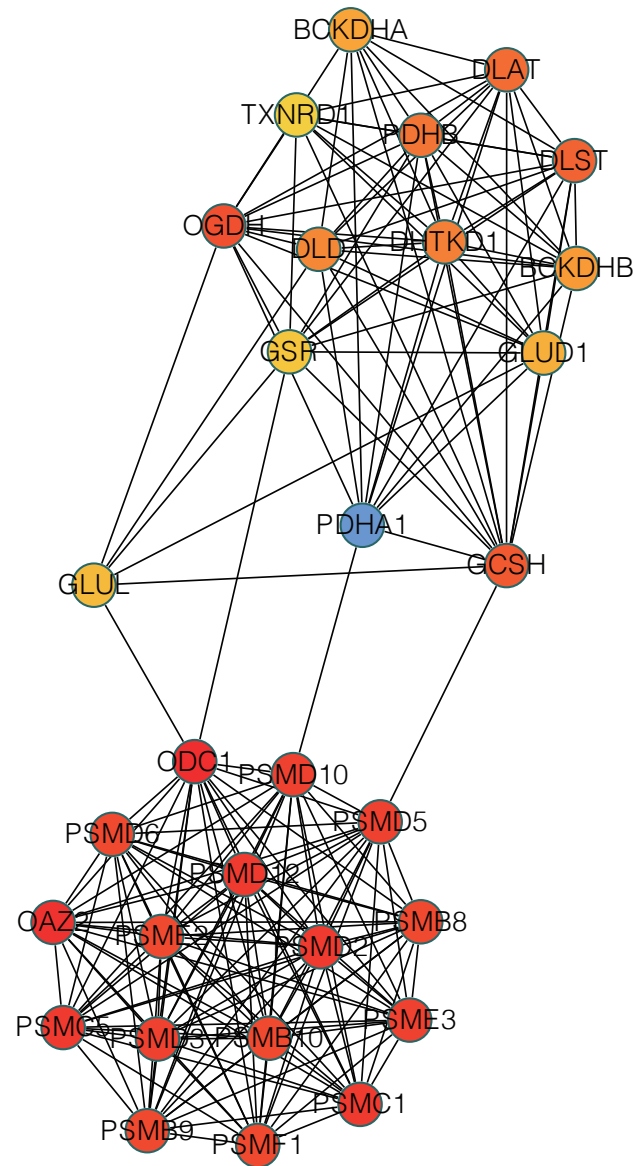

B

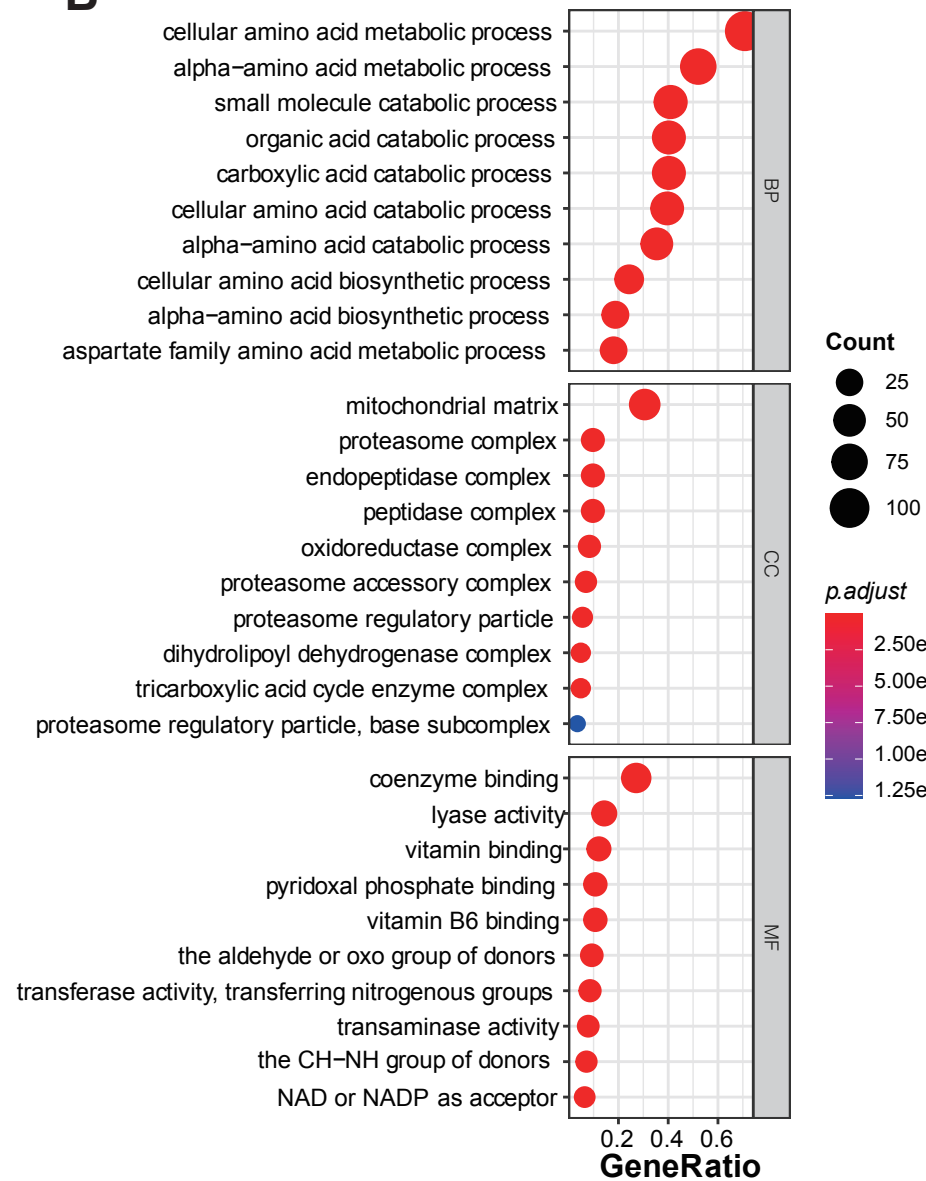

C

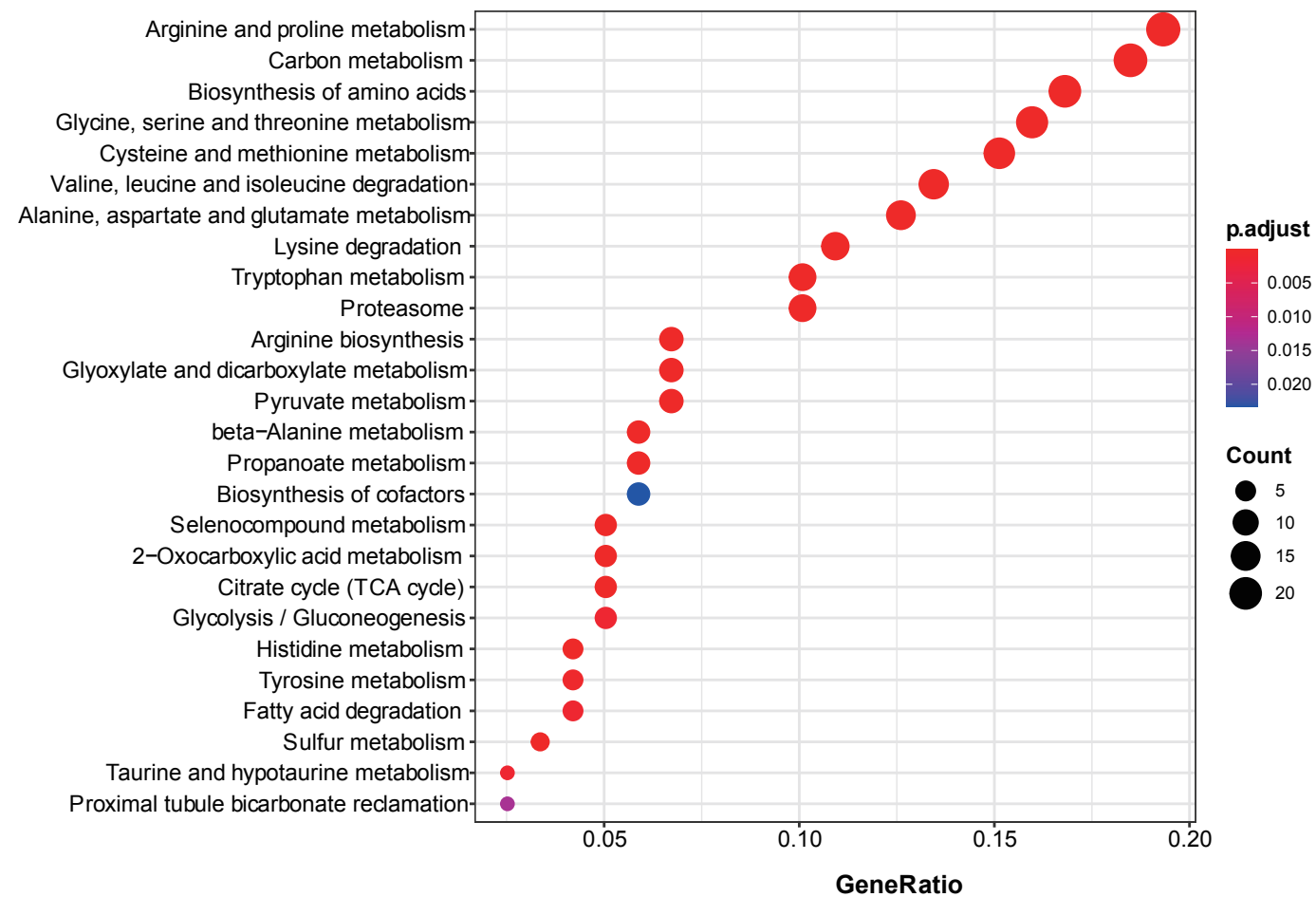

Supplement: Supplementary Figure 3 — Screened out top 10 hub genes of differentially expressed amino metabolism-related genes. (A) The colors of the hub genes were ranked by Maximal Clique Centrality (MCC) value, calculated through Cytoscape plug-in Cytohubba; the depth of the color represents the MCC value. (B) Gene Ontology and Kyoto Encyclopedia of Genes and Genomes analysis of the amino metabolism-related genes. [file DataSheet_3.pdf]

A

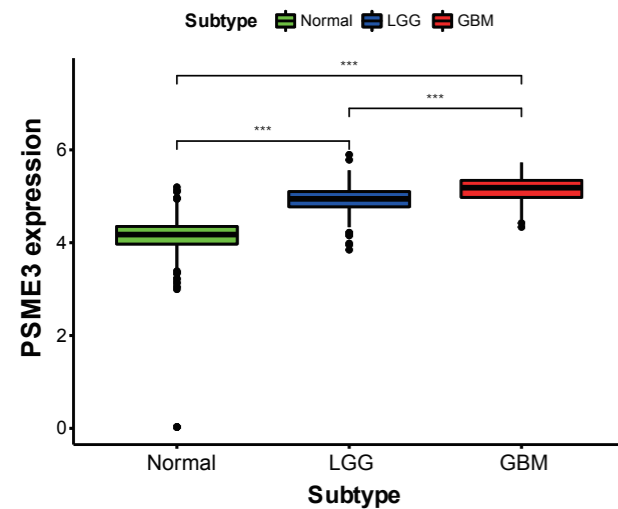

B

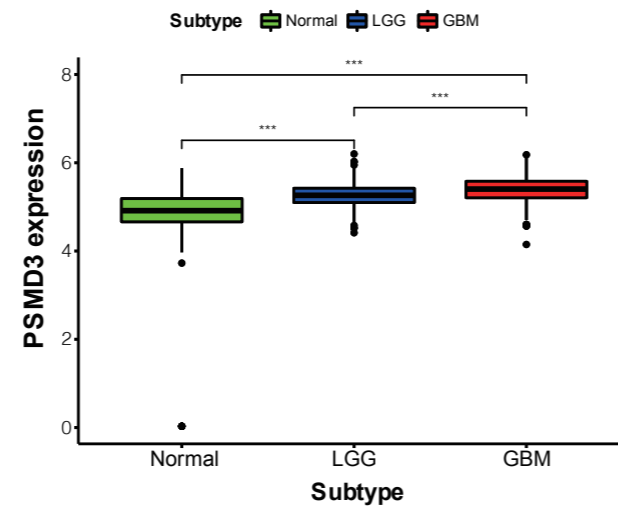

C

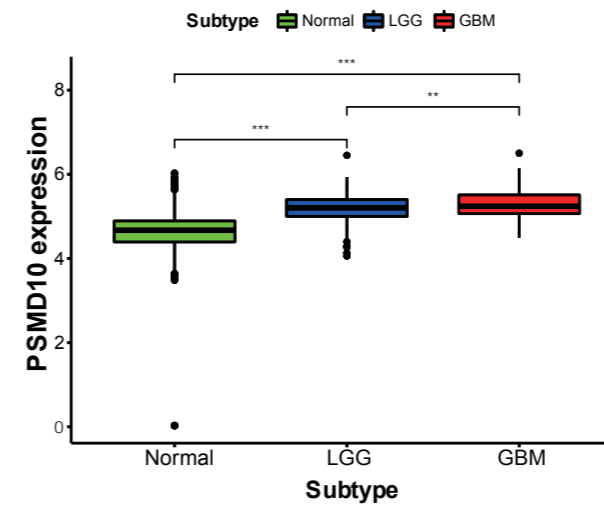

D

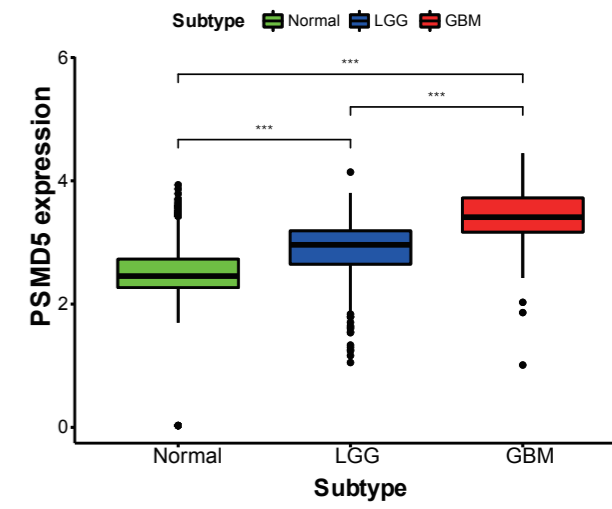

E

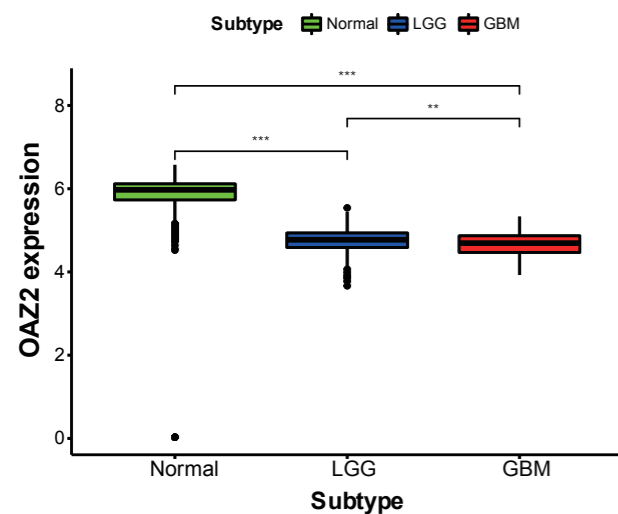

F

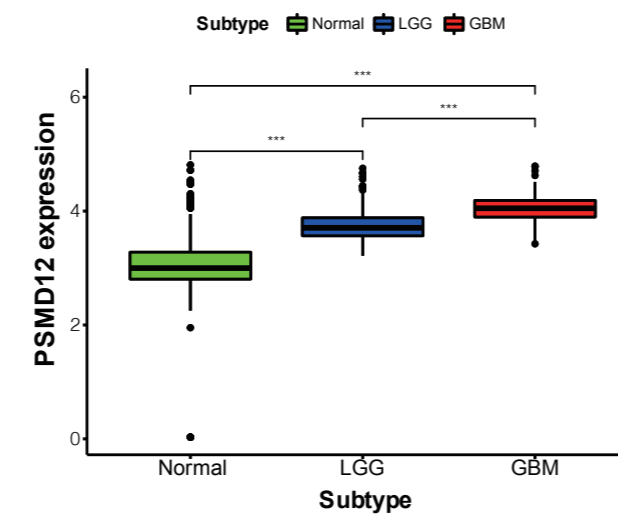

G

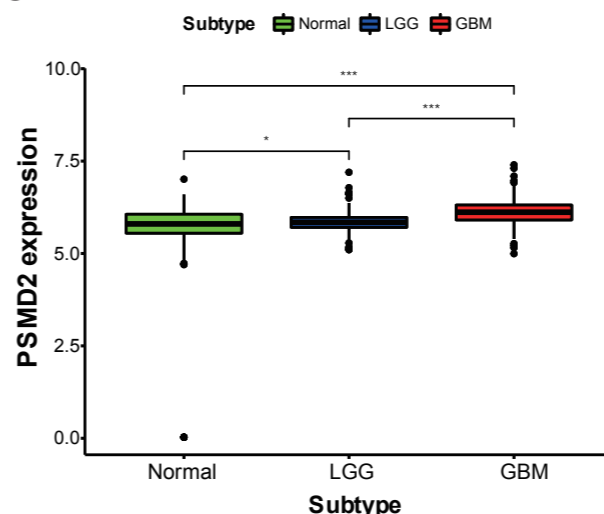

H

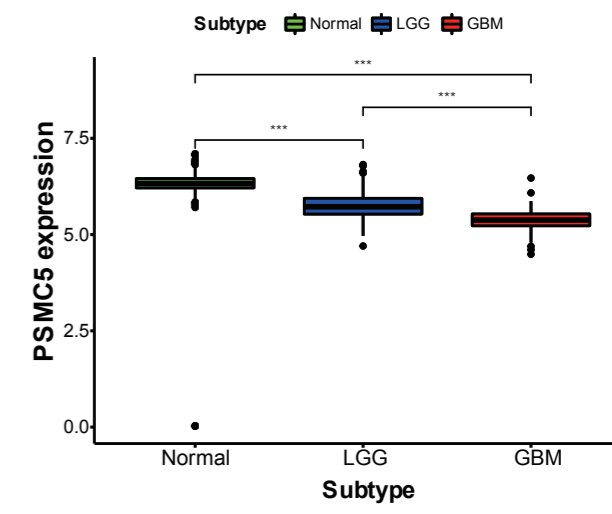

I

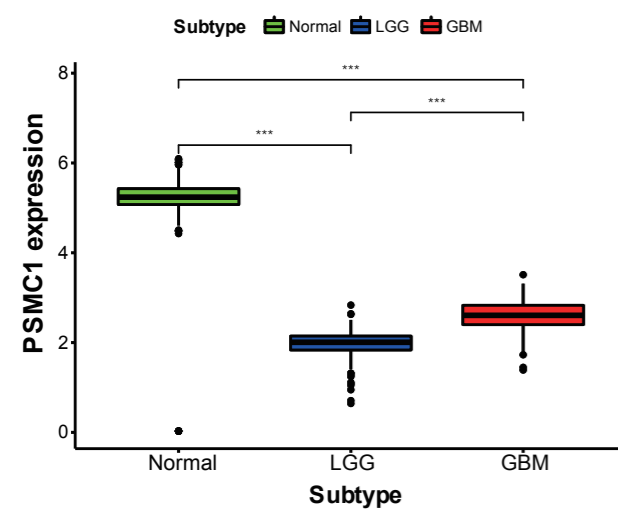

J

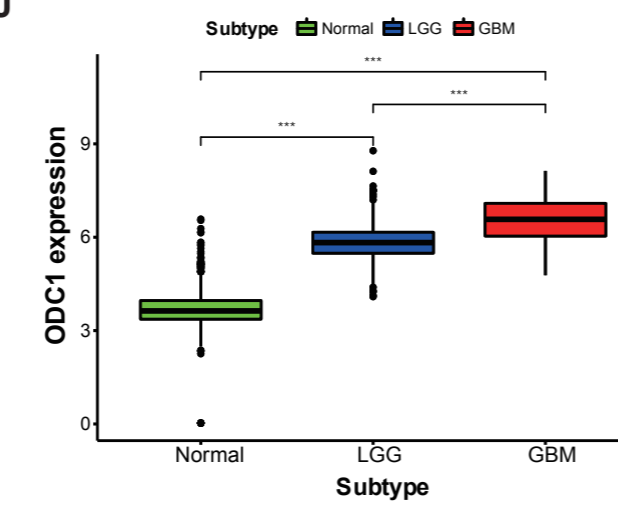

Supplement: Supplementary Figure 4 — The expression level of these 10 hub genes in normal, low-grade glioma (LGG), and glioblastoma (GBM) tissues in the training set. The mRNA expression level of (A) PSME3, (B) PSMD3, (C) PSMD10, (D) PSMD5, (E) OAZ2, (F) PSMD12, (G) PSMD2, (H) PSMC5, (I) PSMC1, and (J) ODC1 in normal, LGG, and GBM tissues in the training set. [file DataSheet_4.pdf]

**A**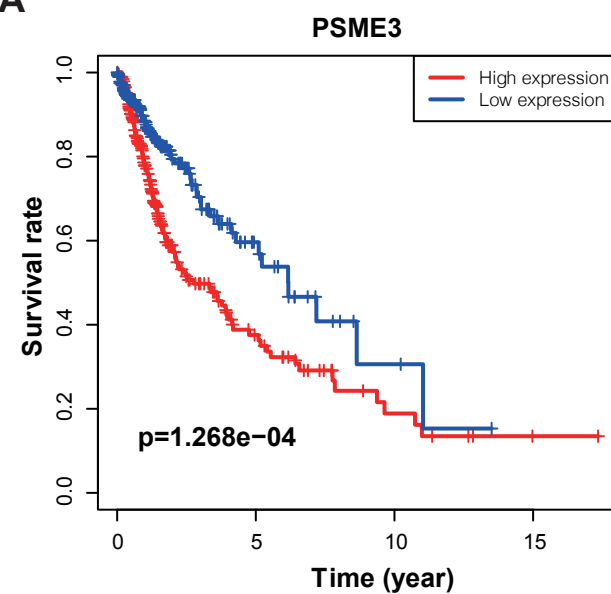**B**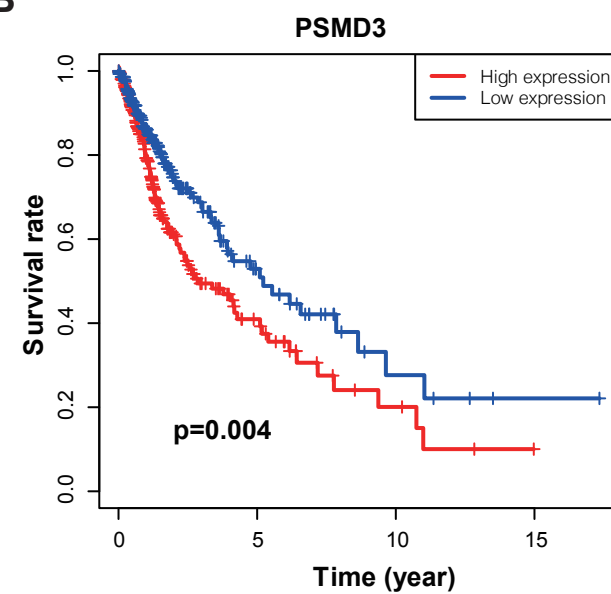**C**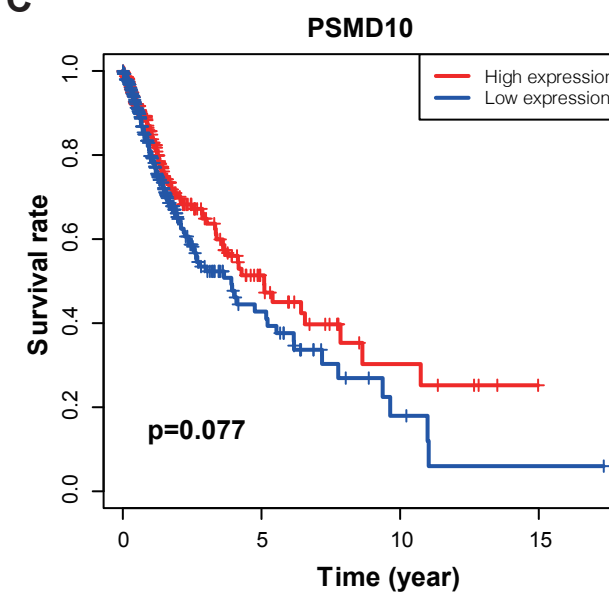**D**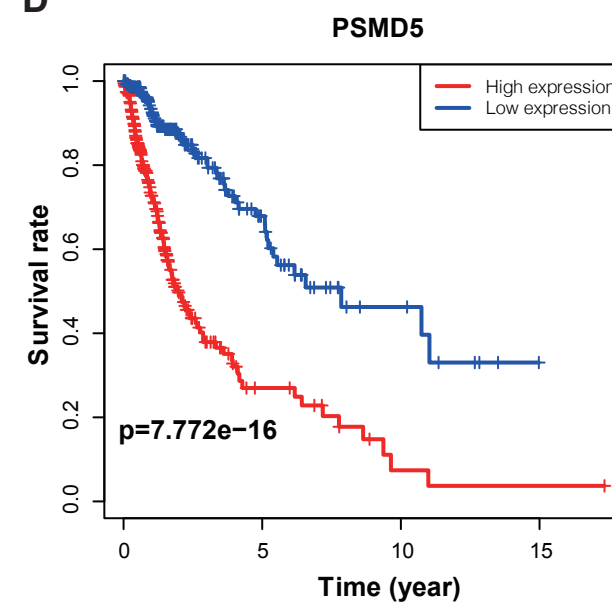**E**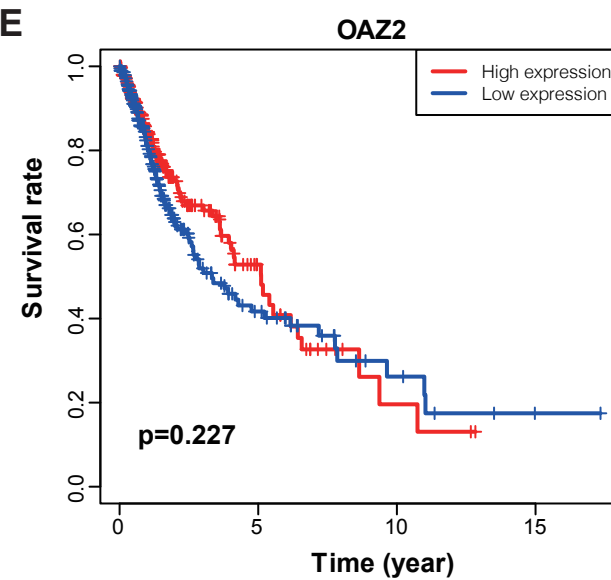**F**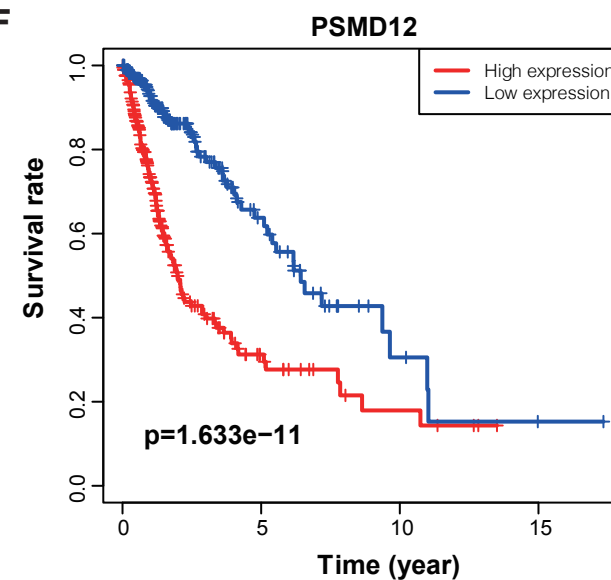**G**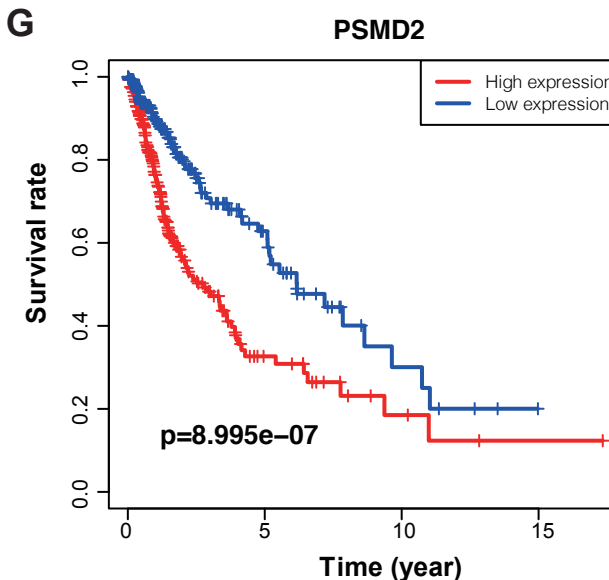**H**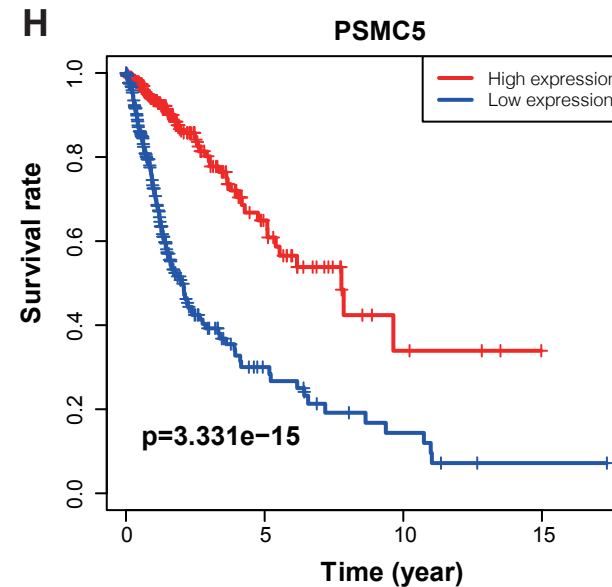**I**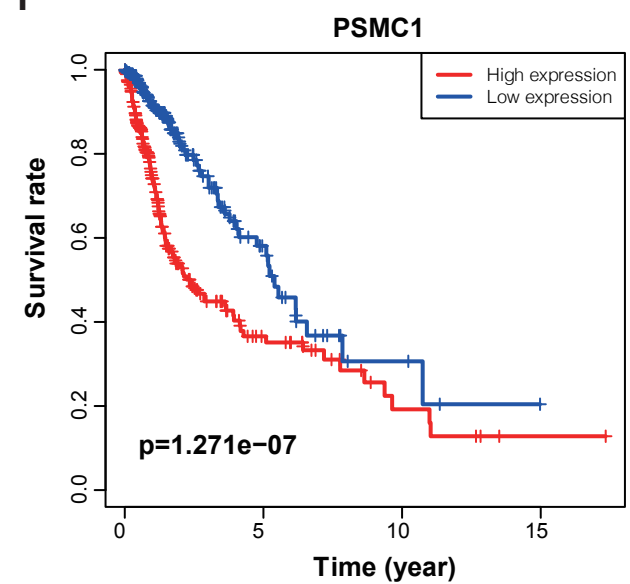**J**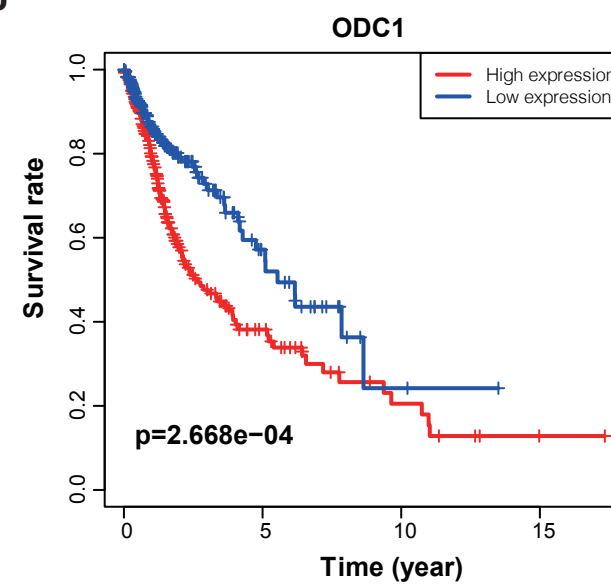

Supplement: Supplementary Figure 5 — The prognostic value of these 10 hub genes in glioma. Kaplan–Meier survival analysis of (A) PSME3, (B) PSMD3, (C) PSMD10, (D) PSMD5, (E) OAZ2, (F) PSMD12, (G) PSMD2, (H) PSMC5, (I) PSMC1, and (J) ODC1 was performed for overall survival in The Cancer Genome Atlas glioma. [file DataSheet_5.pdf]
