# Supplementary material for: Development and Verification of the Amino Metabolism-Related and Immune-Associated Prognosis Signature in Gliomas
Source: Front Oncol. 2021 Nov 5;11:774332. doi: 10.3389/fonc.2021.774332 (PMC8602207; doi:10.3389/fonc.2021.774332)
Supplement: Supplementary file 6 [file DataSheet_6.zip › Supplementary Table 1.docx]

| Top 30 in network network ranked by MCC method | | |
| --- | --- | --- |
| Rank | Name | Score |
| 1 | ODC1 | 1.31E+12 |
| 2 | OAZ2 | 1.31E+12 |
| 3 | PSMD2 | 1.31E+12 |
| 4 | PSMD12 | 1.31E+12 |
| 5 | PSMC1 | 1.31E+12 |
| 6 | PSMC5 | 1.31E+12 |
| 7 | PSMD3 | 1.31E+12 |
| 8 | PSME3 | 1.31E+12 |
| 9 | PSMD10 | 1.31E+12 |
| 10 | PSMD5 | 1.31E+12 |
| 11 | PSME2 | 1.31E+12 |
| 12 | PSMF1 | 1.31E+12 |
| 13 | PSMB10 | 1.31E+12 |
| 14 | PSMB9 | 1.31E+12 |
| 15 | PSMB8 | 1.31E+12 |
| 16 | PSMD6 | 1.31E+12 |
| 17 | OGDH | 552275 |
| 18 | GCSH | 511976 |
| 19 | DLST | 495228 |
| 20 | DLAT | 490610 |
| 21 | PDHB | 489417 |
| 22 | DHTKD1 | 489396 |
| 23 | DLD | 460662 |
| 24 | PDHA1 | 413411 |
| 25 | BCKDHB | 411300 |
| 26 | BCKDHA | 370062 |
| 27 | GLUD1 | 172152 |
| 28 | GLUL | 106271 |
| 29 | GSR | 82584 |
| 30 | TXNRD1 | 81638 |

TableS1. The hub genes calculated by MCC algorithm of CytoHubba plugin
