# Supplementary material for: Development and Verification of the Amino Metabolism-Related and Immune-Associated Prognosis Signature in Gliomas
Source: Front Oncol. 2021 Nov 5;11:774332. doi: 10.3389/fonc.2021.774332 (PMC8602207; doi:10.3389/fonc.2021.774332)
Supplement: Supplementary file 6 [file DataSheet_6.zip › Supplementary Table 2.docx]

|  | TCGA Dataset | | | | | | |  |
| --- | --- | --- | --- | --- | --- | --- | --- | --- |
|  | Univariate analysis | | |  | Multivariate analysis | | |  |
| Variables | **HR** | **95%CI** | **P-value** |  | **HR** | **95%CI** | **P-value** |  |
| Grade | 4.987 | 3.873-6.421 | <0.001 |  | 2.059 | 1.530-2.772 | <0.001 |  |
| Gender | 1.011 | 0.747-1.368 | 0.944 |  | 0.974 | 0.710-1.336 | 0.870 |  |
| Age | 4.863 | 3.391-6.975 | <0.001 |  | 2.296 | 1.465-3.596 | <0.001 |  |
| IDH mutation status | 0.090 | 0.063-0.129 | <0.001 |  | 0.553 | 0.306-1.001 | <0.051 |  |
| 1p19q codeletion status | 0.217 | 0.128-0.370 | <0.001 |  | 0.543 | 0.287-1.025 | 0.060 |  |
| PSME3 | 1.065 | 1.039-1.091 | <0.001 |  | 0.990 | 0.963-1.019 | 0.503 |  |
| PSMD3 | 1.030 | 1.011-1.049 | 0.002 |  | 1.038 | 1.013-1.062 | 0.002 |  |
| PSMD10 | 0.998 | 0.978-1.018 | 0.818 |  | 0.992 | 0.972-1.011 | 0.411 |  |
| PSMD5 | 1.256 | 1.199-1.314 | <0.001 |  | 1.016 | 0.953-1.084 | 0.620 |  |
| OAZ2 | 0.965 | 0.935-0.996 | 0.029 |  | 1.012 | 0.978-1.048 | 0.486 |  |
| PSMD12 | 1.203 | 1.157-1.251 | <0.001 |  | 1.041 | 0.985-1.100 | 0.158 |  |
| PSMD2 | 1.023 | 1.015-1.030 | <0.001 |  | 0.995 | 0.985-1.005 | 0.321 |  |
| PSMC5 | 0.940 | 0.926-0.955 | <0.001 |  | 0.978 | 0.960-0.997 | 0.022 |  |
| PSMC1 | 1.640 | 1.484-1.812 | <0.001 |  | 0.957 | 0.828-1.106 | 0.549 |  |
| ODC1 | 1.007 | 1.005-1.010 | <0.001 |  | 0.997 | 0.993-1.000 | 0.080 |  |

TableS2. Univariate and Multivariate Cox Regression Analyses of Clinicopathologic Characteristics and Hub Genes Associated with Overall Survival in TCGA dataset

Abbreviations: HR, hazard ratio; CI, confidence interval.
