# Supplementary material for: Development and Verification of the Amino Metabolism-Related and Immune-Associated Prognosis Signature in Gliomas
Source: Front Oncol. 2021 Nov 5;11:774332. doi: 10.3389/fonc.2021.774332 (PMC8602207; doi:10.3389/fonc.2021.774332)
Supplement: Supplementary file 6 [file DataSheet_6.zip › Supplementary Table 3.docx]

|  | **Sequences** |
| --- | --- |
| GAPDH Forward | 5′ GGAGCGAGATCCCTCCAAAAT-3′ |
| GAPDH Reverse | 5′-GGCTG TTGTCATACTTCTCATGG-3′ |
| PSMD3 Forward | 5′-CGCCTCAACCACTATGTTCTG-3′ |
| PSMD3 Reverse | 5′-GGACGGAACTGTAAATCAGCC-3′ |
| PSMC5 Forward | 5′-AGGCACAGAGGAACGAACTAA-3′ |
| PSMC5 Reverse | 5′-AGGATGTACCTTGACCAACACTT-3′ |

TableS3. The sequence of primers
